# From Prediction to Function Using Evolutionary Genomics: Human-Specific Ecotypes of *Lactobacillus reuteri* Have Diverse Probiotic Functions

**DOI:** 10.1093/gbe/evu137

**Published:** 2014-06-19

**Authors:** Jennifer K. Spinler, Amrita Sontakke, Emily B. Hollister, Susan F. Venable, Phaik Lyn Oh, Miriam A. Balderas, Delphine M.A. Saulnier, Toni-Ann Mistretta, Sridevi Devaraj, Jens Walter, James Versalovic, Sarah K. Highlander

**Affiliations:** ^1^Texas Children’s Microbiome Center, Department of Pathology, Texas Children’s Hospital, Houston, TX; ^2^Department of Pathology & Immunology, Baylor College of Medicine, Houston, TX; ^3^Department of Food Science and Technology, University of Nebraska, Lincoln; ^4^Department of Molecular Virology & Microbiology, Baylor College of Medicine, Houston, TX; ^5^Human Genome Sequencing Center, Baylor College of Medicine, Houston, TX; ^6^Present address: Department of Gastrointestinal Microbiology, German Institute of Human Nutrition, Nuthetal, Germany.; ^7^Present address: Departments of Agricultural, Food, & Nutritional Science and Biological Sciences, University of Alberta, Edmonton, AB, Canada.; ^8^Present address: Genomic Medicine, J. Craig Venter Institute, La Jolla, CA.

**Keywords:** host-based evolution, reuterin, PocR transcriptional regulation, immunostimulatory, anti-inflammatory, histamine

## Abstract

The vertebrate gut symbiont *Lactobacillus reuteri* has diversified into separate clades reflecting host origin. Strains show evidence of host adaptation, but how host–microbe coevolution influences microbial-derived effects on hosts is poorly understood. Emphasizing human-derived strains of *L. reuteri*, we combined comparative genomic analyses with functional assays to examine variations in host interaction among genetically distinct ecotypes. Within clade II or VI, the genomes of human-derived *L. reuteri* strains are highly conserved in gene content and at the nucleotide level. Nevertheless, they share only 70–90% of total gene content, indicating differences in functional capacity. Human-associated lineages are distinguished by genes related to bacteriophages, vitamin biosynthesis, antimicrobial production, and immunomodulation. Differential production of reuterin, histamine, and folate by 23 clade II and VI strains was demonstrated. These strains also differed with respect to their ability to modulate human cytokine production (tumor necrosis factor, monocyte chemoattractant protein-1, interleukin [IL]-1β, IL-5, IL-7, IL-12, and IL-13) by myeloid cells. Microarray analysis of representative clade II and clade VI strains revealed global regulation of genes within the reuterin, vitamin B_12_, folate, and arginine catabolism gene clusters by the AraC family transcriptional regulator, PocR. Thus, human-derived *L. reuteri* clade II and VI strains are genetically distinct and their differences affect their functional repertoires and probiotic features. These findings highlight the biological impact of microbe:host coevolution and illustrate the functional significance of subspecies differences in the human microbiome. Consideration of host origin and functional differences at the subspecies level may have major impacts on probiotic strain selection and considerations of microbial ecology in mammalian species.

## Introduction

*Lactobacillus reuteri* are natural residents of mammalian and avian gastrointestinal (GI) tracts as well as the human urogenital tract and breast milk. *Lactobacillus reuteri* exhibits strain-specific beneficial properties relevant to human health, making it a model organism for studying host:symbiont interactions as well as microbe:host coevolution. Comparative genomic studies of *Lactobacillus* species performed as part of the Human Microbiome Project highlighted significant heterogeneity within and between species and significant interspecies diversity among strains of *L. reuteri* (Nelson et al. 2010)*.* Previous phylogenetic studies of *L. reuteri* used amplified-fragment length polymorphism and multilocus sequence analysis (MLSA) to study more than 100 strains, and these identified host origin as one basis for intraspecies diversity (Oh et al. 2010), with lineage-specific genomic differences reflecting the niche characteristics in the GI tract of respective hosts (Frese et al. 2011). Experiments in gnotobiotic mice supported host adaptation of *L. reuteri* strains, as only rodent strains colonized mice efficiently (Frese et al. 2011). Human-derived *L. reuteri* strains belong to two distinct MLSA clades, designated clade II and VI. Clade II is remarkably specific to humans and it clusters separately from all other clades, whereas human strains in clade VI are closely related to isolates from chickens (Oh et al. 2010). Overall, the findings indicated that *L. reuteri* is a host-specific symbiont, and separate lineages within the species suggest that host restriction was maintained over evolutionary time spans, allowing host-driven diversification. The nature of evolutionary processes and their influence on specific host-microbe interactions and health status deserve exploration.

The mutualistic relationship between *L. reuteri* and humans has been validated by numerous studies that have documented the ability of *L. reuteri* to elicit probiotic effects in different disorders and disease models. *Lactobacillus reuteri* strains are acid and bile tolerant, produce many of the essential B complex vitamins, notably folate (B_9_) (Santos, Wegkamp, et al. 2008), and cobalamin (B_12_) (Morita et al. 2008; Santos, Vera, et al. 2008), but also potentially thiamin (B_1_) (Saulnier et al. 2011) and riboflavin (B_2_) (Capozzi et al. 2012). Many strains synthesize the antimicrobial compound, reuterin (Axelsson et al. 1989) and the organism synthesizes and secretes the anti-inflammatory biogenic amine, histamine (Thomas et al. 2012). Previous studies examining these probiotic properties and their regulation have indicated that not all strains of *L. reuteri* have equal capacity to express these factors or phenotypes (Lin et al. 2008; Spinler et al. 2008; Jones and Versalovic 2009).

In this study, we compared the genomes of ten sequenced *L. reuteri* strains, derived from three host origins, to gain insight into the distinguishing features of human-derived *L. reuteri* strains, and to better understand the beneficial characteristics specific to human clades II and VI. We have complemented these genomic comparisons with functional data that classify the human-derived strains by gene expression, metabolic function, probiotic features, and effects on cytokine production by human immune cells. These data demonstrate clear differences between the two subpopulations of human-derived probiotic *L. reuteri* strains and emphasize the importance of performing studies on the role of the microbiome in an evolutionary context.

## Materials and Methods

### Bacterial Strains, Human Cell Lines, and Culture Conditions

The human-derived *L. reuteri* strains and genome statistics are listed in table 1. Additional strains used for functional assays are listed in table 2. Bacterial strains were routinely cultured in deMan, Rogosa, Sharpe medium (MRS; Difco, Franklin Lakes, NY) at 37 °C in an anaerobic workstation (MACS MG-500; Microbiology International, Frederick, MD) supplied with a mixture of 10% CO_2_, 10% H_2_, and 80% N_2_ for 16–18 h. Strains ATCC (American Type Culture Collection) 6475::*pocR* and DSM (German Collection of Microorganisms and Cell Cultures) 17938::*pocR* were cultured in the presence of erythromycin (10 µg/ml). Specific culture conditions for individual experiments are detailed throughout. The effect of bacterial supernatants on cytokine production was performed with THP-1 cells (human monocytoid cell line; ATCC TIB-202, ATCC, Manassas, VA) maintained in RPMI (ATCC) and 10% heat-inactivated fetal bovine serum (Invitrogen, Carlsbad, CA) at 37 °C with 5% CO_2_.

**Table 1:** Characteristics of Sequenced *Lactobacillus reuteri* Strains

Strain Name (Alternate)	MLSA Clade^a^	Host (Body Site)	Genome Length (bp)	ORFs	Scaffolds	GenBank Accession Number(s)
JCM 1112	II	Human (GI Tract)	2,039,414	1,820	1	NC_010609
DSM 20016 (F275)	II	Human (GI Tract)	1,999,618	1,900	1	NC_009513
ATCC PTA 4659 (MM2-3)	II	Human (Breast Milk)	1,943,466	2,044	95	NZ_GG693756-6850
ATCC PTA 6475 (MM4-1A)	II	Human (Breast Milk)	2,067,914	2,095	7	NZ_ACGX02000001-007
ATCC 55730 (SD2112)	VI	Human (Breast Milk)	2,264,399	2,246	1	NC_015697
CF48-3A	VI	Human (GI Tract)	2,032,595	2,164	92	NZ_GG693664-755
100-23	III	Rat (GI Tract)	2,305,557	2,181	2	NZ_AAPZ02000001-002
mlc3	III	Mouse (GI Tract)	2,018,630	1,986	126^b^	AEAW00000000
lpuph	I	Mouse (GI Tract)	2,116,621	2,066	127^b^	AEAX00000000
ATCC 53608 (1063)	IV	Pig (GI Tract)	1,968,532	1,864	13	NZ_FR854361-373

^a^As determined by Oh et al. (2010).

^b^Number of contigs.

**Table 2:** Human-Derived *Lactobacillus reuteri* Strains Used in This Study

Strain Name (Alternate)	MLSA Clade^a^	Isolation Site	Source
DSM 20016	II	Feces	DSMZ
ATCC PTA 4659 (MM2-3)	II	Breast Milk	BioGaia AB
ATCC PTA 5289 (FJ1)	II	Oral Cavity	BioGaia AB
ATCC PTA 6475 (MM4-1A)	II	Breast Milk	BioGaia AB
CF15-6	II	Feces	BioGaia AB
CF4-6g	II	Feces	BioGaia AB
JCM 1112	II	Feces	JCM
LMS11-1	II	Feces	MGH
LMS11-3	II	Feces	MGH
MM3-1a	II	Breast Milk	BioGaia AB
SR-11	II	Stomach	O’Toole, PW
SR-14	II	Stomach	O’Toole, PW
ATCC 55730 (SD2112)	VI	Breast Milk	BioGaia AB
CF48-3A	VI	Feces	BioGaia AB
CF6-2a	VI	Feces	BioGaia AB
DSM 17938	VI	Breast Milk	BioGaia AB
M27U15	VI	Breast Milk	BioGaia AB
M45R2	VI	Breast Milk	BioGaia AB
M81R43	VI	Breast Milk	BioGaia AB
MF14-C	VI	Feces	BioGaia AB
MF2-3	VI	Feces	BioGaia AB
MM34-4a	VI	Breast Milk	BioGaia AB
MM36-1a	VI	Breast Milk	BioGaia AB
MV36-2a	VI	Vagina	BioGaia AB
MV4-1a	VI	Vagina	BioGaia AB
ATCC 6475::*pocR*	II	Breast Milk	Santos et al. (2011)
DSM 17938::*pocR*	VI	Breast Milk	This study

Note.—DSMZ, Deutsche Sammlung von Mikroorganismen und Zellkulturen; MGH, Microbiology Laboratories, Massachusetts General Hospital.

^a^As determined by Oh et al. (2010).

### Multilocus Sequence Analysis

MLSA was performed on 119 *L. reuteri* strains. This analysis was completed using standard techniques as previously described (Oh et al. 2010). In brief, seven housekeeping genes (d-alanine-d-alanine ligase [*ddl*], d-alanine-d-alanyl carrier protein ligase [*dltA*], DNA gyrase B subunit [*gyrB*], leucyl-tRNA synthetase [*leuS*], phosphoktolase [*pkt*], recombinase [*recA*], and RNA polymerase alpha subunit [*rpoA*]) were polymerase chain reaction (PCR) amplified from all 119 *L. reuteri* strains, sequenced and included in the MLSA. Clonal Frame software, http://www.xavierdidelot.xtreemhost.com/clonalframe.htm (last accessed July 2, 2014) (Didelot and Falush 2007), which uses a coalescent-based Bayesian method to infer strain relationships, was applied as described previously in supplementary materials of Oh et al. (2010).

### *L. reuteri* Genomes and Pangenome Analysis

Publicly available genome sequences for the ten *L. reuteri* strains (three complete and seven draft sequences) used in this study are described in table 1. Three of the genomes (mlc3, lpuph, and ATCC 53608) lacked annotations so the nucleotide sequences were submitted to the Integrated Microbial Genomes/Expert Review (IMG/ER) system (Markowitz et al. 2010) for gene calling and feature prediction. To maintain consistency, all annotation-based analyses were performed using the IMG annotations for the ten genomes. For draft genomes, we used BLASTz within Advanced PipMaker (Schwartz et al. 2000) to obtain ordered and oriented scaffolds or contigs for comparative analyses. The finished genome of JCM (Japan Collection of Microorganisms) 1112 was used as the reference for clade II, ATCC 55730 for clade VI, 100-23 for mlc3 and lpuph, and I5007 (NC_021494) for ATCC 53608. Once scaffolds or contigs were ordered and oriented, all were arranged so the genome sequence began with the start codon for the *dnaA* gene. These were the nucleotide sequences used for comparative analysis and alignments.

Circular BLASTn-based representations of the ten *L. reuteri* genomes were constructed using BLAST Ring Image Generator (BRIG) (Alikhan et al. 2011). For the incomplete genomes, ATCC 4659, CF48-3A, 100-23, mlc3, lpuph, and ATCC 53608, the ordered and oriented scaffolds were concatenated to create a single scaffold. These and the complete genome nucleotide sequences were used for input in the order shown in figure 2 and supplementary figure S1, Supplementary Material online. The program passes the genomes to BLASTn (release 2.2.28+) using default parameters with the first genome as the reference, and returns a graphical image of concentric rings where the density of color at a particular location within a ring indicates nucleotide homology with respect to the reference.

A pangenome analysis was used to describe the *L. reuteri* species as a whole and to gain insight into the host-driven evolution of the species. Genes in all ten genomes were binned into operational gene units (OGUs) using the online implementation of CD-HIT-EST (Li and Godzik 2006; Huang et al. 2010) with default parameters (>90% nucleotide sequence identity spanning the length of the shorter gene in each pairwise comparison) as described by Hansen et al. (2011). The size of the core genome was estimated utilizing the distribution of shared OGUs among the ten genomes. The function of each OGU was inferred from the annotations obtained from IMG. Higher level functions were obtained from the Kyoto Encyclopedia of Genes and Genomes (KEGG) (Kanehisa et al. 2012) and Cluster of Orthologous Genes (COG) (Tatusov et al. 2003) assignments obtained from the IMG annotation files.

### Average Nucleotide Identity and Gene Conservation between Genome Pairs

We compared the gene conservation at the nucleotide and amino acid levels across the *L. reuteri* genomes to assess diversity within the species. Average nucleotide identity (ANI) and gene conservation between the ten *L. reuteri* genomes were calculated using the BLAST release 2.2.28+ (Altschul et al. 1990), essentially as described previously (Konstantinidis and Tiedje 2005). For ANI, BLASTn was implemented using default parameters except with the drop-off value of 150 for gapped alignments (-xdrop_gap_final 150) and without query filtering/masking (-dust no). ANI was evaluated for all conserved genes having >60% sequence identity over >70% of the length of the query sequence. Gene content was computed using tBLASTn, where predicted protein-coding sequences from one *L. reuteri* genome (designated the query genome) were searched against the genomic sequence of a second *L. reuteri* genome (designated the reference genome), utilizing default settings plus the option –db_gencode 11. The tBLASTn output was filtered by top bit score and *e* value per query, then the query hit was used to score conservation, defined as a coding sequence (CDS) having >60% sequence identity over >70% of the length of the reference CDS.

### Inactivation of *pocR* by Insertional Mutagenesis and Comparative Transcriptional Analysis of ATCC 6475::*pocR* to DSM 17938::*pocR*

The *pocR* gene was inactivated in DSM 17938 as previously described for strain ATCC 6475 (Santos et al. 2011). Briefly, the internal fragment of the *pocR* gene was amplified by PCR using primers RB1883F2 5′-BHI and RB1883R2 3′-ERI (supplementary table S1, Supplementary Material online), and inserted into plasmid pORI28 (Russell and Klaenhammer 2001) by directional cloning using standard techniques (Sambrook and Russell 2001) to generate pORIpocR. Temperature-sensitive, site-specific integration of the nonreplicating plasmid pORIpocR was carried out as described earlier (Russell and Klaenhammer 2001; Walter et al. 2005; Santos et al. 2011). The resulting insertion mutant was designated DSM 17938::*pocR*.

Comparisons of the transcriptome of ATCC 6475::*pocR* and its parent strain were performed using two-color microarrays and published previously (GEO GSE22926; Santos et al. 2011). The transcriptome of the *pocR* mutant strain, DSM 17938::*pocR*, and its parent strain were compared using two-color microarrays as was that of ATCC 6475::*pocR* (Santos et al. 2011). In brief, oligonucleotides were designed and synthesized from a draft genome sequence of *L. reuteri* ATCC 55730 (Saulnier et al. 2011). For expression analysis, stationary phase mRNA was isolated from three biological replicates of the *pocR* mutant and parent strain cultured anaerobically in a semidefined medium, LDMIII (Jones and Versalovic 2009), as described previously (Saulnier et al. 2011). Synthesis, labeling, hybridization, and dye-swap comparisons of cDNA were performed also as previously described (Saulnier et al. 2011). Microarray platform information and data for the DSM 17938 strains are deposited at the National Center for Biotechnology Information (NCBI) Gene Expression Omnibus (GEO GSE54324; http://www.ncbi.nlm.nih.gov/geo/, last accessed July 2, 2014). All image analysis, normalization, and statistical analysis for the DSM 17938 data were performed as before (Santos et al. 2011). Microarray data from the previously published ATCC 6475 data set were compared with the DSM 17938 data set. Only probes common to both arrays were compared.

### Production and Quantification of Reuterin Produced from *L. reuteri* Strains

Isolation of reuterin from *L. reuteri* supernatants was done as previously described (Spinler et al. 2008). Briefly, cell pellets were collected at various time points from anaerobic cultures of *L. reuteri* and were washed in sodium phosphate buffer, resuspended to approximately 1.5 × 10^10^ cells ml^−^^1^ in glycerol solution then incubated anaerobically at 37 °C for 1 h. The reuterin-containing solution was collected, filter sterilized, and stored at 4 °C until further analysis. Reuterin-containing solutions were analyzed colorimetrically by absorbance spectroscopy as done previously (Spinler et al. 2008). A standard curve was generated using 0–6 mM of HPLC quantified reuterin (Spinler et al. 2008) produced by *L. reuteri* DSM 17938.

### Preparation of *L. reuteri* Supernatants

*Lactobacillus reuteri* supernatants were prepared and analyzed for folate and histamine concentrations, and for cytokine bioassays from human monocytes. Strains were cultured anaerobically for 24 h at 37 °C in MRS, and then inoculated into LDMIII at an OD_600_ of 0.1. LDMIII cultures were incubated anaerobically at 37 °C for 24 h, then supernatants were collected, filter sterilized using polyvinylidene fluoride membrane filters (0.22 μm pore size; Millipore, Bedford, MA) and stored at −20 °C before further processing.

### Folate Detection from *L. reuteri* Supernatants

Supernatants of *L. reuteri* strains were prepared as outlined above and tested for the production of folate by electrochemiluminescence (Wilson et al. 2005). Folate levels in the filter-sterilized supernatants were determined by a competitive immunoassay using direct chemiluminescence. Samples were pretreated to release folate conjugates from any endogenous binding proteins in the sample then the free folate was assayed by competition for binding to an acridinium-folate-bound biotin-folate binding protein. Binding was detected using avidin-conjugated paramagnetic particles in the solid phase. The inter- and intra-assay coefficients of variation of the assay were less than 10%.

### Histamine Production by *L. reuteri* Strains and PCR Amplification of *hdcP, hdcA, hdcB,* and *hisRS2* Genes

Supernatants of *L. reuteri* strains were tested for the production of the biogenic amine, histamine. Filter sterilized supernatants were serially diluted in phosphate-buffered saline and tested using a histamine ELISA kit (cat# 409010; Neogen, Lansing, MI) as per the manufacturer’s instructions. Concentrations of histamine were normalized against culture OD_600_ values. PCR amplification was used to confirm the presence or absence of key genes involved in histamine production by *L. reuteri* strains. The sequence of *hisRS2* is sufficiently unique from *hisRS1* as to permit discrimination. Genomic DNA was isolated from *L. reuteri* using the MO BIO Ultra Clean Microbial gDNA isolation kit (Carlsbad, CA) as per manufacturer’s instructions. Target genes were amplified by PCR using standard techniques (Sambrook and Russell 2001), using primers listed in supplementary table S1, Supplementary Material online.

### Bioassay of Cytokine Production from Human Monocytes

*Lactobacillus reuteri* cell-free supernatants were prepared as outlined above and tested for effects on cytokine production by THP-1 cells as previously described (Pena et al. 2004). Supernatants were vacuum-dried, resuspended in RPMI medium, and normalized by volume to OD_600_ = 1.5. THP-1 cells (5 × 10^4^ cells) were stimulated to produce tumor necrosis factor (TNF) by the addition of 100 ng ml^−^^1^ Pam_3_Cys-SKKKK × 3 HCl (EMC Microcollections, Tüebingen, Germany) as previously described (Pena et al. 2004; Lin et al. 2008). *Lactobacillus reuteri* supernatants were added to the activated THP-1 cells (5% v/v) in microtiter plates, which were then incubated at 37 °C in 5% CO_2_ for 3.5 h. THP-1 cells were pelleted (3,000 × g, 5 min, 4 °C), and THP-1 supernatants were assayed using a Human Cytokine/Chemokine-Premixed 14-plex kit (Millipore) in a Luminex 100 system (Luminex Corporation, Austin, TX). The 14 analytes tested were: TNF, granulocyte macrophage colony-stimulating factor, interferon-γ, interleukin (IL)-1β, IL-2, IL-4, IL-5, IL-6, IL-7, IL-8, IL-10, IL-12, IL-13, and monocyte chemoattractant protein (MCP)-1. Raw data were obtained with MasterPlex CT version 1.2.0.7 and analyzed with MasterPlex QT version 5.0.0.73 (Hitachi MiraiBio, San Francisco, CA).

### Statistical Analysis of Microbiological Data

Statistical analyses were performed using SPSS 19.0 (IBM SPSS Statisticsm; http://www.01.ibm.com/software/analytics/spss/, last accessed July 2, 2014). Data reported are means from a minimum of three biological replicates for reuterin, histamine, folate, and cytokine assays. Conditions were defined as negative control, positive control, clade II, or clade VI. A one-way analysis of variance (ANOVA) was run between conditions to generate *P* values. A Tukey HSD (Honestly Significant Difference) algorithm was applied to the ANOVA result to reduce the Type I error rate (false positives). All significance values are based on *P* < 0.05. Folate data were analyzed using the Student’s *t*-test with one-tailed distribution and considered statistically significant at a *P* value ≤ 0.05, unless otherwise stated.

## Results and Discussion

### General Features of *L. reuteri* Genomes

**Fig. 1.—:**
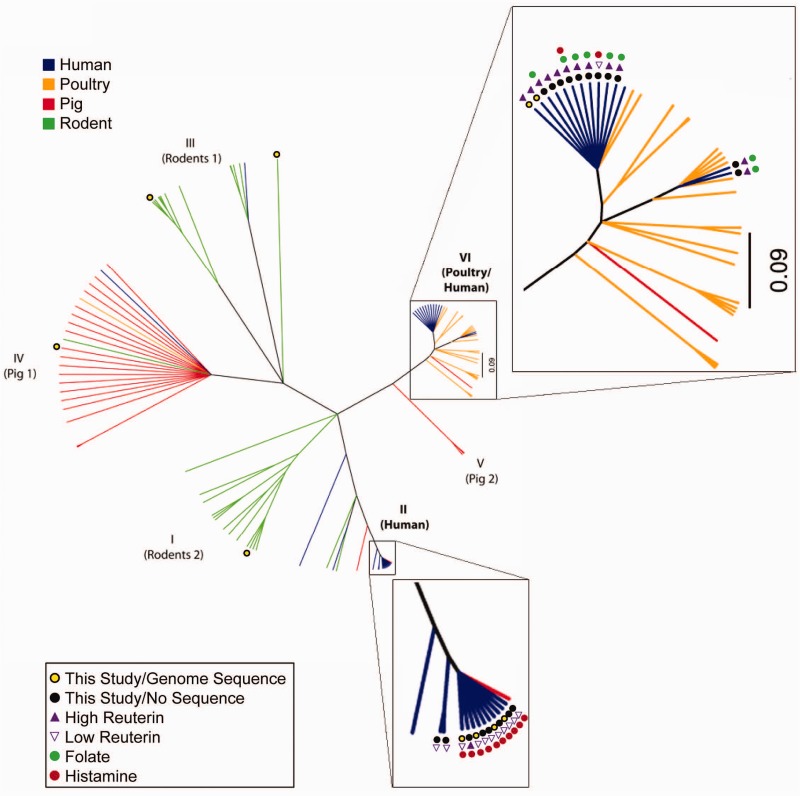
Phylogenetic analysis of *Lactobacillus reuteri* isolates derived from four hosts. The genealogy of 119 *L. reuteri* strains based on MLSA of sequences. Tree branches are color coded by host origin: Green = rodent, red = pig, blue = human, yellow = poultry. The 25 strains included in the functional analyses are indicated by closed black circles 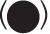, and strains with sequenced genomes are indicated by a closed black circle with a yellow center 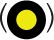. Reuterin production is designated as follows: 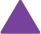 high reuterin production, 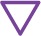 low reuterin production. Strains that produce high amounts of folate are indicated by closed green circles 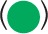, and strains producing histamine are indicated by closed red circles 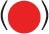.

**Fig. 2.—:**
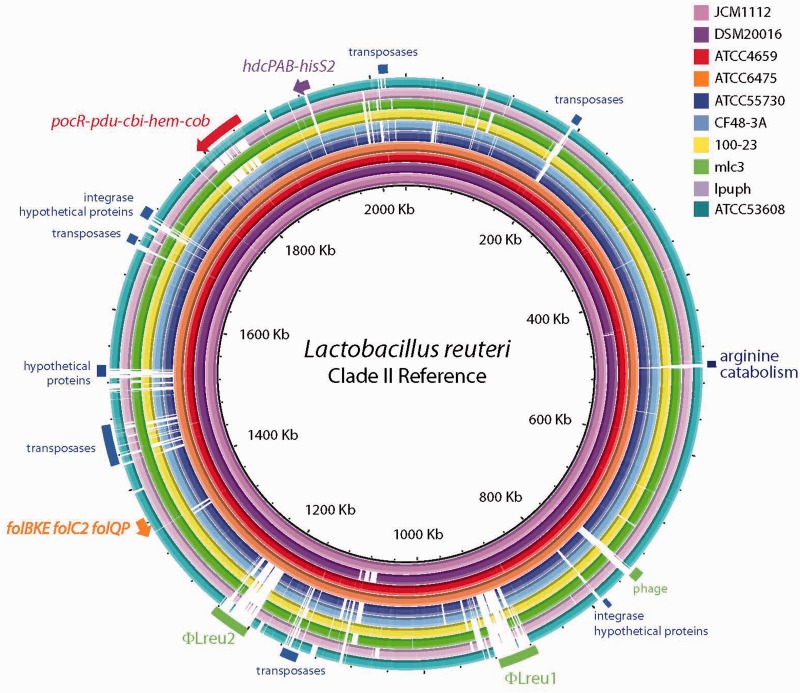
Genomic comparison of ten *Lactobacillus reuteri* genomes to clade II strain JCM 1112. Diagram represents BLASTn results of each genome against JCM 1112 with results rendered using the BRIG program (Alikhan et al. 2011). Each genome is color coded as indicated by the legend. Relative shading density (from darker to lighter) within each circle represents relative levels of nucleotide homology. White regions indicate regions with no identity to the reference. Features of interest are annotated.

As of June 2013, the genomes of ten *L. reuteri* strains had been sequenced (three complete genomes and seven draft sequences), and their features are described in table 1. Additional *L. reuteri* genomes of strains I5007 and TD1 were deposited in NCBI during the preparation of this manuscript, but were not included in this analysis. The genomes of the strains analyzed here are similar in size (1.9–2.3 Mb), G+C content (38.4–39.0%), and contain between 1,820 and 2,300 reported predicted protein-coding sequences. The *L. reuteri* strains are isolates from three host types (human, rodent, and pig) and were collected from four geographically distinct regions within Australasia, Europe, North America, and South America. *Lactobacillus reuteri* is grouped into host-associated clades based on MLSA typing (fig. 1, table 1), and the ten genomes were assigned to clades I–IV and VI by Oh et al. (2010), with six genomes from human-derived strains belonging to clades II and VI. To visualize differences between the genomes, we generated the nucleotide alignments of the genomes shown in figure 2 by BLASTn using the complete clade II genome (JCM 1112) as the reference and the program BRIG (Alikhan et al. 2011). We also created a clade VI reference alignment, using the completed genome of ATCC 55730 as the reference, which is shown in supplementary figure S1, Supplementary Material online.

We conducted a pangenome analysis to better understand the distinct and independent evolution of the *L. reuteri* strains. All predicted protein-coding genes present in these genomes (∼20,600 genes) were binned into OGUs with other genes having >90% identity using the program CD-HIT (Huang et al. 2010). The predicted pangenome contains approximately 3,700 OGUs, and the number of new OGUs accumulating within the pangenome began to plateau after the addition of eight *L. reuteri* genomes (∼16,500 CDSs), indicating its approach to saturation. The rate and shape of the rarefaction curve (data not shown) predicts that the pangenome should close with approximately 24 *L. reuteri* genomes. The overall conserved genome is approximately 1.2 Mb, which represents about 1,230 core OGUs. We also identified 1,200 unique genes in *L. reuteri*. Strain-specific differences are defined by those genes within the pangenome which are not shared among strains. Although these genes are referred to as “unique,” this designation is context-dependent, since it is possible that they may be found in other bacterial species. Finally, approximately 1,320 members of the pangenome are dispensable genes were identified, which are neither unique genes nor members of the core genome.

### Examining Ecological and Evolutionary Diversity within *L. reuteri*

**Fig. 3.—:**
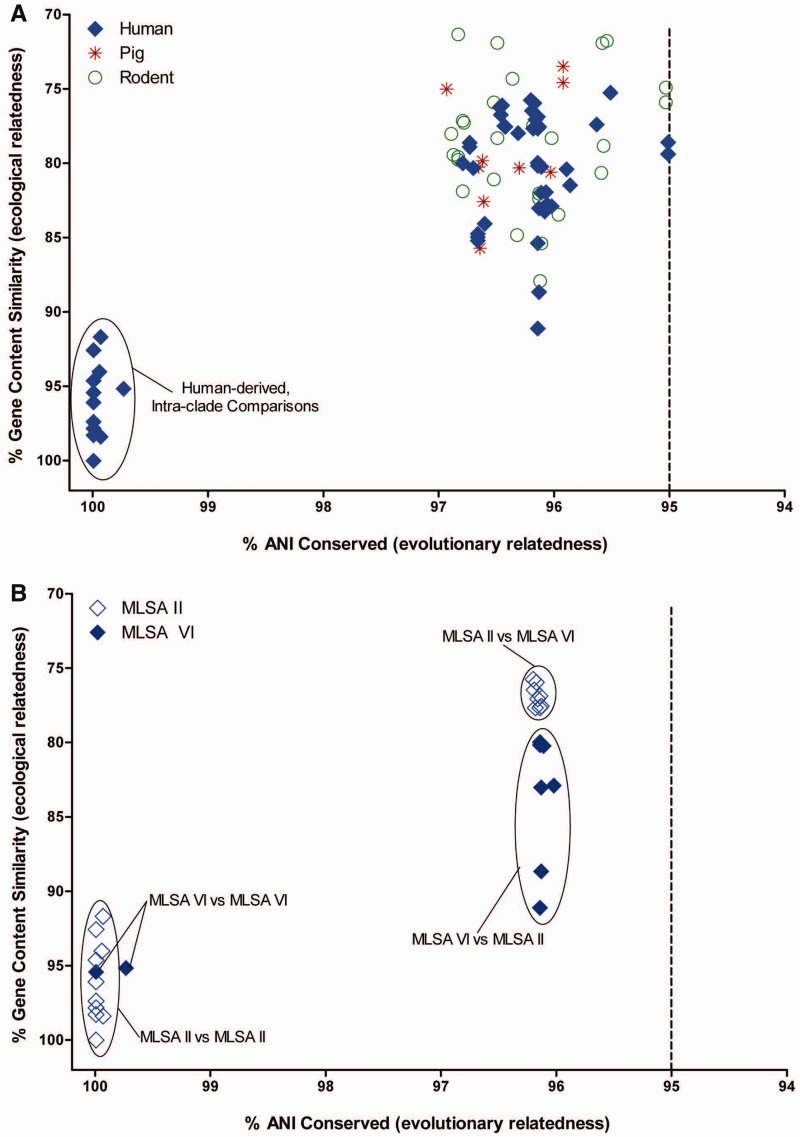
Ecological and genetic diversity within the *Lactobacillus reuteri* species. Conserved genes (*y* axis) versus average nucleotide evolutionary distance (*x* axis) plot for ten sequenced *L. reuteri* genomes isolated from three mammalian hosts. Each data point represents a whole-genome comparison between two genomes. (*A*) Pairwise comparisons between all ten genomes. Intraclade comparisons of human-derived strains are circled near the origin. (*B*) Pairwise comparisons of human-derived MLSA II and MLSA VI *L. reuteri* genomes.

To explore patterns of evolution and ecology within *L. reuteri*, pairwise genomic comparisons of the ANI of each predicted CDS were compared with the amino acid similarity (gene content) of each CDS (fig. 3). Higher percentage values of both ANI and gene content suggest that genomes have evolved similarly and inhabit comparable niches, whereas lower percentage values indicate genomes that have evolved divergently and adapted to different environments (Konstantinidis et al. 2006). The ten-genome pairwise comparison revealed two distinct populations within the *L. reuteri* species and confirmed similar intraspecies diversity revealed earlier with a seven-genome comparison reported by Nelson et al. (2010). Despite having been isolated from disparate hosts, all clades shared ANIs of 95% or greater. Utilizing a bacterial species definition of 70% DNA–DNA hybridization (Wayne 1987) or ≥95% ANI (Konstantinidis and Tiedje 2005), the intraspecies divergence observed among our strains does not dictate a new species designation as all pairwise comparisons have ≥95% ANI (fig. 3*A*). Of note, the two human-derived clades were as dissimilar to one another as they were to clades that contained rodent or porcine-derived strains, suggesting that the two human clades may have evolved separately. Conversely, within-clade ANI values exceed 99% and gene content levels were 92–100% suggesting that genomes within clades II and VI may have undergone clade-specific clonal diversification.

The population structure and the distinct genomic features of *L. reuteri* lineages indicate that these lineages may have been shaped by different evolutionary forces, and we hypothesized that metabolic functions and probiotic phenotypes would differ among the two human-derived *L. reuteri* ecotypes. Here we consider the definition of an ecotype to represent a cohesive group that possesses all the dynamic properties attributed to a species, but one that remains irreversibly separate and ecologically distinct (Cohan and Koeppel 2008). Our findings confirm extremely low sequence variation among strains within the same human ecotype. By MLSA analysis, human-derived *L. reuteri* clade VI strains cluster closely with strains of poultry origin, whereas clade II strains almost exclusively associate with other human-derived strains (fig. 1). Additionally, our pangenome analysis showed that the majority of the genes that distinguished strains in clades II and VI encode hypothetical proteins (data not shown). These observations are similar to those from *Bacillus anthracis* and *Salmonella enterica* pathovar Typhi genome projects, where strains were considered identical because of >99% ANI, but where further examination revealed the acquisition of a small number of genetic elements capable of delineating distinct ecotypes (Konstantinidis and Tiedje 2005). In this study, we made predictions of function based on genome content using genome sequences of six human-derived *L. reuteri* strains that represent clades II and VI (see fig. 2 and supplementary fig. S1, Supplementary Material online). To validate the functional differences defining the two human-derived ecotypes, we expanded our study to analyze the clade-specificity of probiotic-associated traits of 25 human-derived *L. reuteri* strains representing clades II (12 strains) and clade VI (13 strains) (table 2; fig. 1).

### The Presence of Clade-Specific Mobile Genetic Elements Support Clonal Evolution of Human-Derived *L. reuteri* Strains

Mobile genetic elements (such as insertion elements, transposons, and genes associated with bacteriophage) contribute to host specialization of *L. reuteri* (Frese et al. 2011), and accounted for an additional 126 OGUs specific to human-derived strains. Two complete prophages, unique to the clade II genomes, are clearly identified in figure 2. We assigned new open reading frames (ORFs) and updated the annotations (supplementary tables S2*a* and *b*, Supplementary Material online) as described in the Materials and Methods. ϕLreu1 is about 54 kb in length and is 100% identical among the clade II genomes. It is inserted between a gluconate transporter and the riboflavin synthase alpha and beta subunits, and its structural proteins are most similar to other temperate *Siphoviridae* proteins from *L. plantarum* genomes. The second prophage, ϕLreu2, is about 43.5 kb long, also is 100% conserved and lies between the 50S large subunit protein gene *rpsL* and the beta-galactosidase operon. It contains a potential self-splicing intron element at its terminus. The ϕLreu2 structural proteins suggest that it may also be a member of the *Siphoviridae*, as its capsid, portal and head-tail joining proteins are very similar to those of phage HK97 family phages*.* The clade VI strains also contain two complete prophages (ϕLreu3 and ϕLreu4) that were revealed on the BLASTn-based map using ATCC 55730 as the reference genome (supplementary fig. S1, Supplementary Material online). The annotations for these, also likely *Siphoviridae*, are listed in supplementary tables S2*c* and *d*, Supplementary Material online. Many transposase genes are present in the genomes, though their types and distributions vary between clades II and VI. Most notably, all of the sequenced clade II genomes carry 19 copies of the IS*200*/IS*605* family transposase gene whereas the clade VI genomes have only two. This IS*200*/IS*605* family of insertion elements is of interest because they are the smallest known, lack characteristic inverted repeats, and insert in a site-specific manner (Barabas et al. 2008). The 100% identity and clade-specific delineation of bacteriophages and transposase genes in these genomes corroborate the notion that the human-derived clade II and VI genomes are distinct clonal groups, and further support the idea that human-derived *L. reuteri* strains have undergone a clade-specific selective sweep important for adapting to the human GI tract (Walter et al. 2011).

### Distinct Metabolic Functions and Probiotic Phenotypes Have Evolved to Define the Two Human-Derived *L. reuteri* Ecotypes

Probiotic *L. reuteri* produce factors that are beneficial to the mammalian host, aid in host defense, and help to maintain a homeostatic relationship in the gut. Many strains synthesize the antimicrobial compound, reuterin (Axelsson et al. 1989), and specific strains synthesize and secrete the anti-inflammatory biogenic amine, histamine (Thomas et al. 2012); each of which will be discussed in detail later. Additionally, lactic acid bacteria survive local acidic environments and synthesize essential B complex vitamins.

*Lactobacillus reuteri* has an arginine catabolism mechanism that may function to facilitate survival at low pH (Marquis et al. 1987). An approximately 27 kb gene cluster, which is flanked by an IS*200* insertion element and maps between 484,793 and 511,393 in JCM 1112, carries core and dispensable genes that are observed in multiple clades but have a specific gene order and composition in clades II and VI (fig. 2); updated annotations for these are presented in supplementary table S3, Supplementary Material online. The region contains 31 ORFs and carries the *arc* genes responsible for producing enzymes that catabolize arginine to citrulline and ornithine and ultimately to ammonia, thereby providing a means for *L. reuteri* to thrive in the presence of low pH in the lactic acid environment or during gut transit.

*Lactobacillus reuteri* produce many essential B complex vitamins (Morita et al. 2008; Santos, Vera, et al. 2008; Santos, Wegkamp, et al. 2008; Saulnier et al. 2011; Capozzi et al. 2012). Folate (vitamin B_9_), specifically, is formed from the precursor molecules, 6-hydroxymethyl-7,8-dihydropterin pyrophosphate (DHPPP) and para-aminobenzoic acid (pABA). Most lactobacilli encode the enzymes required to generate folate from DHPPP and pABA, but not those required for pABA synthesis (Rossi et al. 2011); thus, they are not predicted to perform de novo synthesis of folate (supplementary fig. S2, Supplementary Material online). Unexpectedly, many of the clade VI *L. reuteri* strains tested do produce folate in the presence of limiting concentrations of pABA, whereas folate production by clade II strains was not observed under these conditions (supplementary fig. S2, Supplementary Material online). The organization of the folate genes is similar in both clades, and the proteins have high sequence homology (93–99%). The *folB*, *folK*, *folE*, *thfs* (*folC2*), *folQ,* and *folP* genes are clustered and map between nucleotides 1,363,448 and 1,369,044 in JCM 1112 (fig. 2 and supplementary table S4, Supplementary Material online); *folA* and *folC1* are single genes that map at other positions within the genome (supplementary table S4, Supplementary Material online).

### Production of the Antimicrobial Compound, Reuterin, Is Enhanced in *L. reuteri* Clade VI Strains

**Fig. 4.—:**
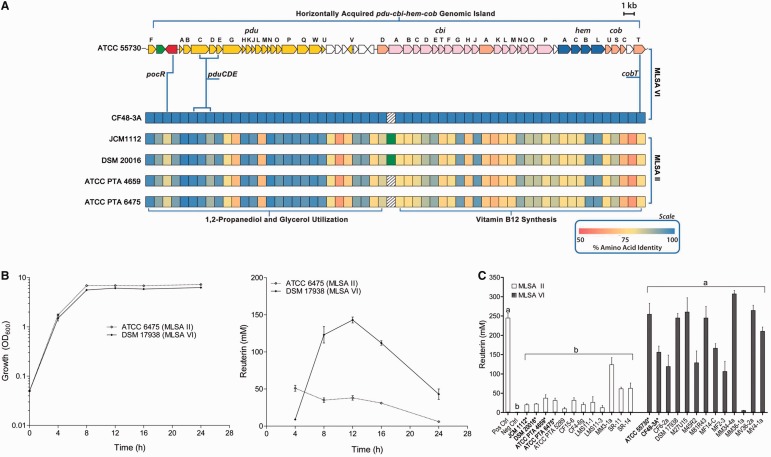
The *pdu–cbi–hem–cob* gene cluster and clade-specific reuterin production. (*A*) CDS map of the 57 gene *pdu–cbi–hem–cob* cluster that encodes the PocR regulator (shown in red), genes *pduCDE* responsible for production of the antimicrobial reuterin, Pdu proteins required for 1,2-propanediol and glycerol utilization, and Cbi, Hem, and Cob proteins required for synthesis of vitamin B_12_, as per Morita et al. (2008). Below the CDS map are heat maps representing percent amino acid identities of each protein within the *pdu–cbi–hem–cob* gene cluster of four MLSA II *Lactobacillus reuteri* genomes relative to the amino acid sequences of MLSA VI proteins from strains ATCC 55730 and CF48-3A. (*B*) Growth curves of strains ATCC 6475 (clade II) and DSM 17938 (clade VI) in MRS medium at 37 °C under anaerobic conditions (left); growth phase production of reuterin by strain ATCC 6475 (clade II) and DSM 17938 (clade VI) (right). (*C*) Reuterin production at stationary phase (12 h) from 25 human-derived *L. reuteri* strains. Strains with sequenced genomes are indicated in bold with an asterisk (*). Results are expressed as the mean ± SD, *n* = 3, and significant differences between group means as determined by one-way ANOVA (*P* < 0.05) are indicated by the placement of different letters above each bracketed group.

Most human-derived isolates of *L. reuteri* produce the broad-spectrum antimicrobial compound, reuterin (a mixture of 3-hydroxypropionaldehyde isomers) (Vollenweider et al. 2003; Walter et al. 2011), from glycerol, in a vitamin B_12_-dependent process (Axelsson et al. 1989; Talarico and Dobrogosz 1990). Reuterin production is dependent on the presence of the horizontally acquired 57 gene cluster, *pdu–cbi–hem–cob* (fig. 4*A* and supplementary table S5, Supplementary Material online) (Morita et al. 2008), which contains the *pdu* genes that encode enzymes required for glycerol and 1,2-propanediol utilization, linked to the *cbi–hem–cob* genes that encode the proteins required for the biosynthesis of vitamin B_12_, or cobalamin (Santos, Vera, et al. 2008). In previous work, we observed that strain ATCC 55730 (clade VI) produced 3-fold more reuterin than the clade II strains ATCC 6475, ATCC 4659, and ATCC 5679 during the stationary phase of growth (Spinler et al. 2008). To further examine the strain-specific characteristics of reuterin production, we tested whether reuterin production by human-derived *L. reuteri* was growth phase dependent.

We compared reuterin production in clade VI strain DSM 17938 (a plasmid-cured version of ATCC 55730; Rosander et al. 2008) and ATCC 6475 (clade II), at time points representing logarithmic (4 h), early stationary (8 h), stationary (12 h), and late stationary (24 h) growth phases (fig. 4*B*). In *L. reuteri* strain DSM 17938, reuterin production increased more than 16-fold from approximately 9 mM during logarithmic phase (4 h) to approximately 140 mM during stationary phase (12 h; fig. 4*B*). In contrast, in strain ATCC 6475, reuterin production was growth phase independent until late stationary phase and was about one-third that of DSM 17938 (fig. 4*B*). To expand these observations, we measured in vitro reuterin production from stationary phase cultures of the 23 additional human-derived *L. reuteri* strains. Statistically significant (*P* < 0.05) clade-specific differences were observed with all but one clade VI strain (MM36-1a). With the exception of this strain, all clade VI strains produced >100 mM reuterin, whereas all but three of the clade II strains produced less than 50 mM reuterin (fig. 4*C*).

Although the organization of the *pdu–cbi–hem–cob* gene cluster is conserved in all *L. reuteri* genomes that carry it, the genomic islands have distinct clade-specific sequence homology (fig. 2 and supplementary fig. S1, Supplementary Material online). A comparison of the amino acid identity of each predicted ORF from the human strains relative to the corresponding ORF in ATCC 55730 is shown in figure 4*A*. Note that all of the CF48-3A protein sequences are 100% identical to those of ATCC 55730. In clade II, high levels of homology between clades (>95%) are restricted to two of the three reuterin biosynthesis proteins (PduC and PduD) plus additional proteins encoded by the *pdu* operon. The predicted vitamin B_12_ biosynthesis proteins encoded by the *pdu–cbi–hem–cob* genomic island are not as similar between clades (61–95% identity) (fig. 4*A*). The glycerol dehydratase requires vitamin B_12_ as a cofactor for activity, so differences in vitamin B_12_ biosynthetic capacity should also impact the organism’s capacity to produce reuterin.

### Clade-Specific Transcriptional Regulation by the AraC-Like Regulator, PocR

Just as pathogenic microbes possess stringent mechanisms for regulating virulence factors, beneficial microbes have evolved specific mechanisms for regulating probiotic functions. A gene encoding one such regulator, PocR, which is a member of the AraC family of transcriptional regulators, is encoded by the *pdu–cbi–hem–cob* gene cluster (Morita et al. 2008). The 364 amino acid PocR protein encoded by ATCC 55730 and CF48-3A is 100% identical; however, the clade VI and clade II proteins are only 80% identical (fig. 4*A*). In a previous study comparing RNA expression levels from wild-type and a *pocR* insertion mutation in ATCC 6475, we showed that PocR regulates transcription of operons involved in both reuterin and vitamin B_12_ synthesis in the clade II strains JCM 1112 and ATCC 6475 (Santos et al. 2011). The growth phase-dependent production of reuterin observed in clade VI strain DSM 17938 suggested that its production might be transcriptionally regulated. To test this, we created an equivalent *pocR* insertion mutation in DSM 17938 and performed comparative microarray analysis. Inactivation of *pocR* in either ATCC 6475 or DSM 17938 affected expression of genes not only within the *pdu–cbi–hem–cob* gene cluster as anticipated (supplementary fig. S3 and tables S6 and S7, Supplementary Material online), but also at other loci relevant to probiosis, including genes involved in arginine catabolism and folate production, as listed in supplementary tables S6 and S7, Supplementary Material online. Although not part of this study, it should be noted that we previously were able to complement the ATCC 6475::*pocR* mutation with a plasmid-borne copy of the ATCC 6475 *pocR* gene (Santos et al. 2011) so the insertions are likely nonpolar.

When comparing PocR-affected gene expression, the most striking strain-dependent difference was observed for *pocR* itself. PocR is predicted to function by both activating and repressing transcription from cognate promoters (Santos et al. 2011), and as an AraC family transcriptional regulator it may also regulate its own transcription. Because the *pocR* mutations were gene interruptions, not deletions, it was possible to score *pocR* transcription in the mutants. Under the conditions tested, DSM 17938 (clade VI) PocR did not affect its own expression; however in ATCC 6475, *pocR* transcription was repressed approximately 38-fold, suggesting that the protein is auto-regulatory in this clade II strain (supplementary fig. S3 and tables S6 and S7, Supplementary Material online). These results, along with others (Rondon and Escalante-Semerena 1992; Chen et al. 1994; Santos 2008; Mellin et al. 2013), indicate that strain-specific regulatory mechanisms can exist within human-derived *L. reuteri*.

Strain-dependent transcriptional regulation by PocR is observed for the major regulons of the 57 gene *pdu–cbi–hem–cob* cluster (supplementary fig. S3, Supplementary Material online). As discussed, the *pdu* genes encode distinct functions including the production of the antimicrobial compound, reuterin (Talarico and Dobrogosz 1990; Morita et al. 2008) and the utilization of glycerol and propanediol as electron acceptors to support growth (Talarico et al. 1990; Sriramulu et al. 2008). This is in contrast to other organisms that have two isofunctional vitamin B_12_-dependent enzymes for dehydrating glycerol or propanediol (Toraya and Fukui 1977; Toraya et al. 1980; Daniel et al. 1998). As the PduCDE enzyme is vitamin B_12_ dependent, upregulation of the *cbi–hem–cob* genes along with the *pdu* genes, as observed in strain DSM 17938 (clade VI), is consistent with elevated reuterin production by this clade (fig. 4). Conversely, the low level of expression of *cbi–hem–cob* genes in ATCC 6475, even in the presence of high levels of *pdu* expression, may explain the reduced reuterin production in this clade (supplementary fig. S3, Supplementary Material online). Overall, these data suggest that the human-derived strains from clades II and VI differentially exploit the functionality of the *pdu*–*cbi–hem–cob* cluster, with the former focusing this cluster on energy gain whereas the latter produces more reuterin.

### Factors Produced by *L. reuteri* Strains Differentially Affect Cytokine Production by Human Myeloid Cells

We quantified 11 human cytokines produced by stimulated THP-1 monocytoid cells in response to treatment with cell-free supernatants from 12 clade II and 13 clade VI strains (table 3). The effects were generally clade-specific and consistent with the prior literature regarding *L. reuteri* and immunomodulation (Pena et al. 2005). Clade II strains can be considered anti-inflammatory based on aggregate cytokine responses (table 3) and the established correlation between TNF inhibition and suppression of intestinal inflammation by *L. reuteri* (Pena et al. 2005; Hemarajata et al. 2013). In addition to suppressing TNF, most clade II strains suppressed MCP-1, IL-1β, and IL-12. Suppression of the proinflammatory cytokines TNF, MCP-1, IL-1β, and IL-12 by clade II strains is consistent with the known ability of *L. reuteri* strain ATCC 6475 to suppress intestinal inflammation.

**Table 3:** The Effects of *Lactobacillus reuteri* Secreted Factors on Cytokine Profiles of Human Immune Cells

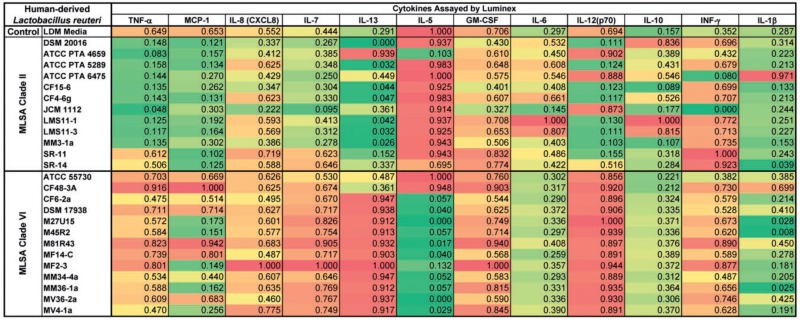

Note.—Inflammatory cytokine production by Toll-like receptor 2-activated THP-1 cells in the presence of culture supernatants from *L. reuteri* clade II or clade VI strains. Supernatants from THP-1 cells were assayed using a Human Cytokine/Chemokine-Premixed 14-plex kit in a Luminex 100 system. All values are presented on a 0–1 scale, indicating a max/min normalization per analyte, and are displayed as heatmaps with red indicating values closer to the maximum (1) and green indicating values closer to minimum (0).

In contrast to the suppression of proinflammatory cytokines by clade II, supernatants derived from clade VI strains stimulated IL-7, IL-12, and IL-13 production (*P* < 0.05 vs. media control and clade II strains, table 3). Clade VI strains are considered immunostimulatory based on these aggregate cytokine responses and they may serve the host by promoting the development and maintenance of an active mucosal immune surveillance system. Most clade VI members (but not ATCC 55730 and CF48-3A) suppressed IL-5 production (table 3); these findings appear to be inconsistent with the aggregate cytokine responses. However, IL-5 can play a prominent role at sites distal to the intestine and may modulate immune responses at other body sites. IL-5 is a cytokine that promotes eosinophil differentiation from bone marrow precursor cells, and acts as a potent eosinophil chemoattractant (Akuthota and Weller 2012). Eosinophilic inflammation contributes to distinct disease phenotypes of the skin, lungs, and sinuses, and suppression of eosinophilic inflammation could be beneficial in decreasing atopic and allergic diseases away from the intestine. Oral administration of *L. reuteri* ATCC 55730 (clade VI) can modulate IFN-γ and IL-4 cytokine expression at sites distal from the intestine in patients with atopic dermatitis (Miniello et al. 2010), and can reduce the incidence of IgE-associated eczema at 2 years of age (Abrahamsson et al. 2007; Forsberg et al. 2013). Others have shown that oral administration of *L. reuteri* modulates allergic airway responses in mice (Forsythe et al. 2007; Karimi et al. 2009).

### Histamine Production by *L. reuteri* Clade II Strains Corresponds with Anti-Inflammatory Properties

**Fig. 5.—:**
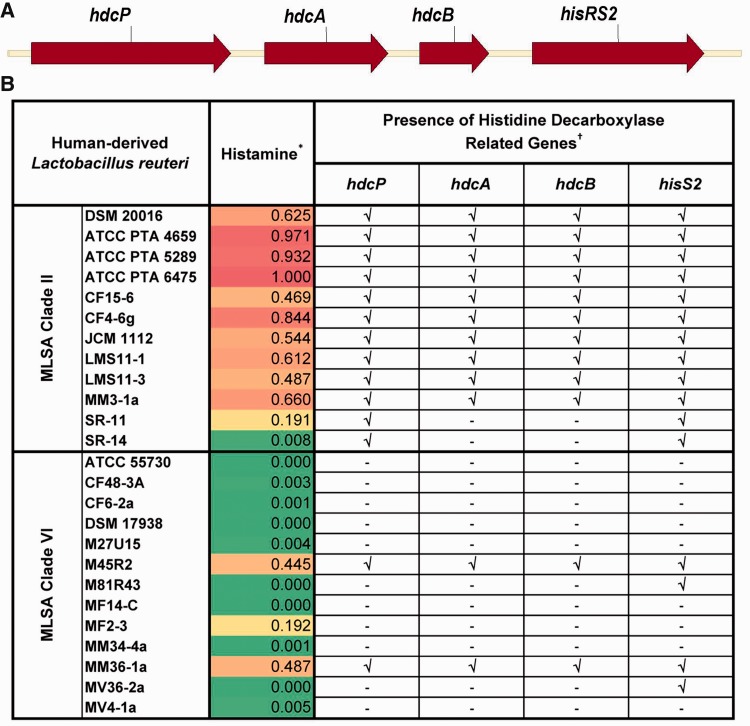
Relative production of histamine by human-derived *Lactobacillus reuteri* clades. (*A*) CDS map of the *hdcP–hdcA–hdcB–hisRS2* gene cluster in clade II *L. reuteri* strains. All values are presented on a 0–1 scale, indicating a max/min normalization per analyte, and are displayed as heatmaps with red indicating values closer to the maximum (1) and green indicating values closer to minimum (0). (*B*) Histamine in conditioned media by 25 human-derived *L. reuteri* strains was measured by ELISA and compared with media control. Results of end-point PCR tests for the four histidine decarboxylase cluster genes are summarized: √ indicates correct PCR product observed; - indicates no PCR product observed.

The anti-inflammatory properties of *L. reuteri* ATCC 6475 (clade II) have been extensively characterized, and some have recently been linked to its ability to produce histamine from histidine (Thomas et al. 2012; Hemarajata et al. 2013). In *L. reuteri,* and other lactobacilli, histamine is produced by a pyruvoyl-dependent histidine decarboxylase (HdcA) (Thomas et al. 2012). The *hdcA* gene is linked to *hdcB*, encoding a protein of unknown function that is presumed, by association, to have a role in the decarboxylation system (van Poelje and Snell 1990; Le Jeune et al. 1995). A gene encoding a histidine–histamine antiporter (*hdcP*) (Lucas et al. 2005) lies immediately upstream of *hdcAB* in the clade II strains and it is directly linked to a second histidyl-tRNA synthetase (*hisRS2*) gene that shares limited homology with the core *hisRS* gene (fig. 5*A*). The predicted HisRS2 protein contains the catalytic residues required for histidyl-tRNA synthesis (Sissler et al. 1999) so it is likely that this protein contributes to the pool of charged tRNAs available for protein synthesis under conditions of histidine depletion in this organism, which is a histidine auxotroph.

In JCM 1112, the histidine decarboxylase locus maps between coordinates 1923821 and 1928862 (supplementary table S8, Supplementary Material online) and it occurs at similar chromosomal positions in the genomes of DSM 20016, ATCC 4659, and ATCC 6475, but the genes are not present in the genomes of ATCC 55730 or CF48-3A. PCR analysis of additional nonsequenced clade VI strains revealed the occasional presence of the gene cluster in this clade (see below). Database searches revealed the presence of complete histidine decarboxylase gene clusters on *Lactobacillus* plasmids pLRI01 (GenBank accession number NC_021504), pLF01 (GenBank accession number AVAB01000110), and an unstable 80 kb plasmid in *L. hilgardii* IOEB 0006 (Lucas et al. 2005); as well as in several *Lactobacillus* genomes: *L. buchneri* B301 (Martin et al. 2005), *L. saerimneri* 30a (Romano et al. 2013), *L. sakei* LTH 2076 (GenBank accession number DQ13288), and *L. vaginalis* ATCC 49540 (GenBank accession number NZ_ACGV0). The histidine decarboxylase gene (*hdcA*) is also found in the incomplete genomes of *L. fructivorans* KCTC 3543 (GenBank accession number NZ_AEQY) and *L. rossiae* DSM 15814 (GenBank accession number NZ_AUAW).

Bioassays of the 25 human-derived *L. reuteri* strains show that the majority of clade II strains produced histamine (fig. 5*B*), and conditioned media from histamine producers suppressed TNF production from human monocytes by more than 50% relative to controls (table 3). All *L. reuteri* strains, including those in clade VI, were tested by PCR for the presence of the *hdcP, hdcA, hdcB*, and *hisRS2* genes (fig. 5*B*). As expected, all histamine producing strains in both clades contained the entire gene cluster and nonproducing strains lacked the histidine decarboxylase gene and *hdcB.* Clade II strains SR-11 and SR-14 were *hdcP^+^hdcA^−^hdcB^−^hisRS2^+^* suggesting that they may have incurred an internal deletion within the gene cluster; the presence of this deletion was not confirmed. Clade VI strains M81R43 and MV36-2a both yielded a positive signal for the *hisRS2* gene but were negative for the remainder of the histidine decarboxylase gene cluster. Two clade VI strains, M45R2 and MM36-1a, are *hdcP^+^hdcA^+^hdcB^+^hisRS2^+^* and produce histamine (fig. 5*B*), but do not suppress TNF production (table 3). Perhaps, these strains produce histamine antagonists or other factors that counteract this anti-inflammatory phenotype. Although histamine production by *L. reuteri* clade II strains has clearly been linked to the TNF-suppressive phenotype, other clade specific factors may be responsible for the effects on additional anti-inflammatory cytokines observed here.

### Do Distinct Probiotic Functions Reflect the Evolutionary History of Strains?

Our analyses demonstrate that strains of clade II and VI differ substantially in their functional attributes, which is likely to reflect their distinct ecology and the symbiotic relationships that they maintain with their hosts. Currently, we can only speculate on how these differences evolved. The majority of clade II isolates were isolated from human fecal samples, and these strains do not cluster with isolates from other host species (Oh et al. 2010), suggesting that clade II represents the autochthonous *L. reuteri* population in the human intestinal tract. In contrast, in clade VI, human isolates cluster tightly with isolates from poultry (Oh et al. 2010), and as strains from this cluster are extremely rare in human fecal samples (but very common in poultry fecal samples), they might be allochthonous to humans originating from poultry (Oh et al. 2010; Frese et al. 2011). Alternatively, if we consider that the majority of human-derived clade VI strains are indeed autochthonous to humans, the population genetic structure suggests that migration to humans was very recent. In either case, it is likely that clade VI strains have evolved with poultry for most of their evolutionary history. Our findings support such distinct evolutionary trajectories.

The ecological niches occupied by *L. reuteri* strains in the GI tracts of chickens and humans are highly distinct. *Lactobacillus reuteri* is one of the most abundant *Lactobacillus* spp. in the crop of chickens where it forms biofilms on the stratified squamous epithelium of the crop (Abbas Hilmi et al. 2007). Accordingly, genome comparisons by microarray analysis revealed that genome content of clade VI strains is more similar to rodent and pig strains, reflecting similarities in lifestyle, probably related to similarities in biofilm formation in the proximal GI tract of these animals. In contrast, the human GI tracts do not contain stratified squamous epithelia, and *Lactobacillus* biofilms have not been demonstrated in the human gut (Walter 2008). This is consistent with the genome content of clade II strains, which have deleted virtually all large surface proteins annotated as adhesins and the two exopolysaccharide clusters present in *L. reuteri* strains from rodents (supplementary fig. S1, Supplementary Material online; Frese et al. 2011).

Following the theory that clade II strains are autochthonous to humans, they might focus the function of the PduCDE enzyme on propanediol utilization and energy generation to support growth in the human gut where simple sugars are limited. Propanediol is produced by gut microbes as a fermentation byproduct of rhamnose and fucose (Badia et al. 1985). These simple sugars are components of glycoconjugates found in intestinal epithelial cells and plant cell walls (Bry et al. 1996), as well as in breast milk oligosaccharides (Castanys-Munoz et al. 2013). Sriramulu et al. (2008) have shown that DSM 20016 (clade II) shows a growth advantage over rodent-derived *L. reuteri* 100-23 (that lacks the *pdu–cbi–hem–cob* cluster) when propanediol is provided in the growth medium, further supporting the idea that clade II strains preferentially utilize the propanediol dehydratase activity of PduCDE.

Furthermore, the cytokine bioassays reveal that most clade II strains are associated with suppression of proinflammatory cytokines produced by macrophages, whereas most clade VI strains are associated with a general proinflammatory cytokine increase, and specifically suppress IL-5. Clade VI strains may play an important role in fostering development and maintenance of robust and fully mature immune responses by the gut microbiome. Clade II strains may serve an equally important role in helping the host regulate and resolve immune responses, and suppress potentially deleterious effects of mucosal inflammation. As the immune cells used in our assays are of human origin, the findings might reflect the distinct relationships of the clades with the human host.

## Conclusions

**Fig. 6.—:**
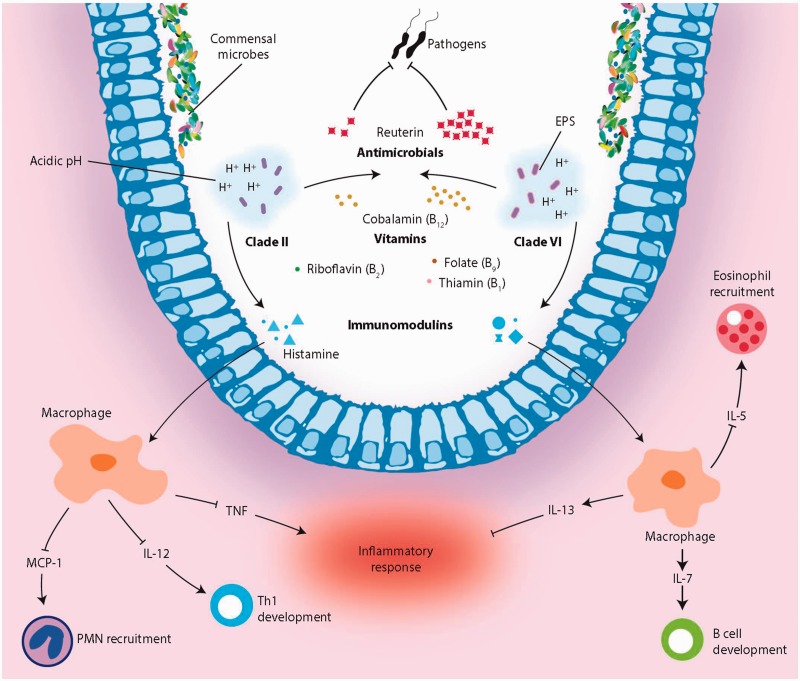
Functional illustration of human-derived *Lactobacillus reuteri* ecotypes. *Lactobacillus reuteri* strains from both clades modulate acidic environments, produce the antimicrobial reuterin, synthesize essential vitamins, and generate immunomodulatory compounds that effect immune signaling in the host. The differences associated with each ecotype are illustrated here.

Host-driven evolution of *L. reuteri* has resulted in ecotypes that specialize toward particular host species (Oh et al. 2010; Frese et al. 2011). We show that human strains from two distinct ecotypes differ markedly in bacterial functions that are likely to influence the interrelationships of these strains with the human host (fig. 6), indicating that the evolutionary trajectory of a gut microbe influences bacterial traits that can be beneficial to its host. The most striking differences between the two ecotypes are the amounts of the antimicrobial compound reuterin produced, and the remarkable differences in intestinal immunomodulatory capacity between the clades. Clade II strains can be considered immunosuppressive and clade VI strains can be considered immunostimulatory. The contrasting immunomodulatory effects of these two separate clades provide a prominent example how different lineages within a single bacterial species can benefit the host by different mechanisms. The different genomic characteristics of clades II and VI evident in the ANI and gene content analysis (fig. 3*B*) indicate that the evolution of the two human-derived *L. reuteri* ecotypes was influenced by separate and distinct environmental pressures (and potentially by two different hosts). Most importantly, the evolutionary process impacted how strains interact with host cells. Thus, our work provides novel information about host–microbe interrelationships as it shows that distinct evolutionary paths within the same species are likely to determine how gut microbes impact their host, which is likely important for their health effects. Important lineage distinctions within a single bacterial species have recently been described in other organisms and include the distinction of two different clades of *Wolbachia* infecting the same population of *Drosophila* (Ellegaard et al. 2013)*,* subclades of freshwater isolates of the Alphaproteobacteria SAR11 that are unique and distinct from saltwater clades (Zaremba-Niedzwiedzka et al. 2013), and the identification of four distinct clades of *Chlamydia trachomatis* associated with four distinct disease types (Joseph et al. 2012). These and our work are concrete examples of the evolution of discreet ecotypes within a bacterial species and address the “bacterial species concept” (Rossello-Mora and Amann 2001).

These findings suggest that evolutionary criteria could be helpful for the selection of probiotic strains. Understanding the genomic basis of the probiotic characteristics associated with distinct lineages of *L. reuteri* will ultimately allow us to predict and assign groups of probiotic *L. reuteri* strains best suited to prevent or treat various classes of diseases.

## Supplementary Material

Supplementary figures S1–S3 and tables S1–S8 are available at *Genome Biology and Evolution* online (http://www.gbe.oxfordjournals.org/).

Supplementary Data
