# Supplementary material for: From Prediction to Function Using Evolutionary Genomics: Human-Specific Ecotypes of Lactobacillus reuteri Have Diverse Probiotic Functions
Source: Genome Biol Evol. 2014 Jun 19;6(7):1772–89. doi: 10.1093/gbe/evu137 (PMC4122935; doi:10.1093/gbe/evu137)
Supplement: Supplementary Data [file supp_6_7_1772__index.html]

From prediction to function using evolutionary genomics: Human-specific ecotypes of Lactobacillus reuteri have diverse probiotic functions — From Prediction to Function Using Evolutionary Genomics: Human-Specific Ecotypes of Lactobacillus reuteri Have Diverse Probiotic Functions — Supplementary Data 

# From Prediction to Function Using Evolutionary Genomics: Human-Specific Ecotypes of *Lactobacillus reuteri* Have Diverse Probiotic Functions

## Supplementary Data

files

**Files in this Data Supplement:**

- Supplementary Data - jpg file
- Supplementary Data - jpg file
- Supplementary Data - jpg file
- Supplementary Data - docx file
- Supplementary Data - docx file
- Supplementary Data - docx file
- Supplementary Data - docx file
- Supplementary Data - docx file
- Supplementary Data - xlsx file
- Supplementary Data - xlsx file
- Supplementary Data - docx file
