# Supplementary figures and images for: From Prediction to Function Using Evolutionary Genomics: Human-Specific Ecotypes of Lactobacillus reuteri Have Diverse Probiotic Functions
Source: Genome Biol Evol. 2014 Jun 19;6(7):1772–89. doi: 10.1093/gbe/evu137 (PMC4122935; doi:10.1093/gbe/evu137)

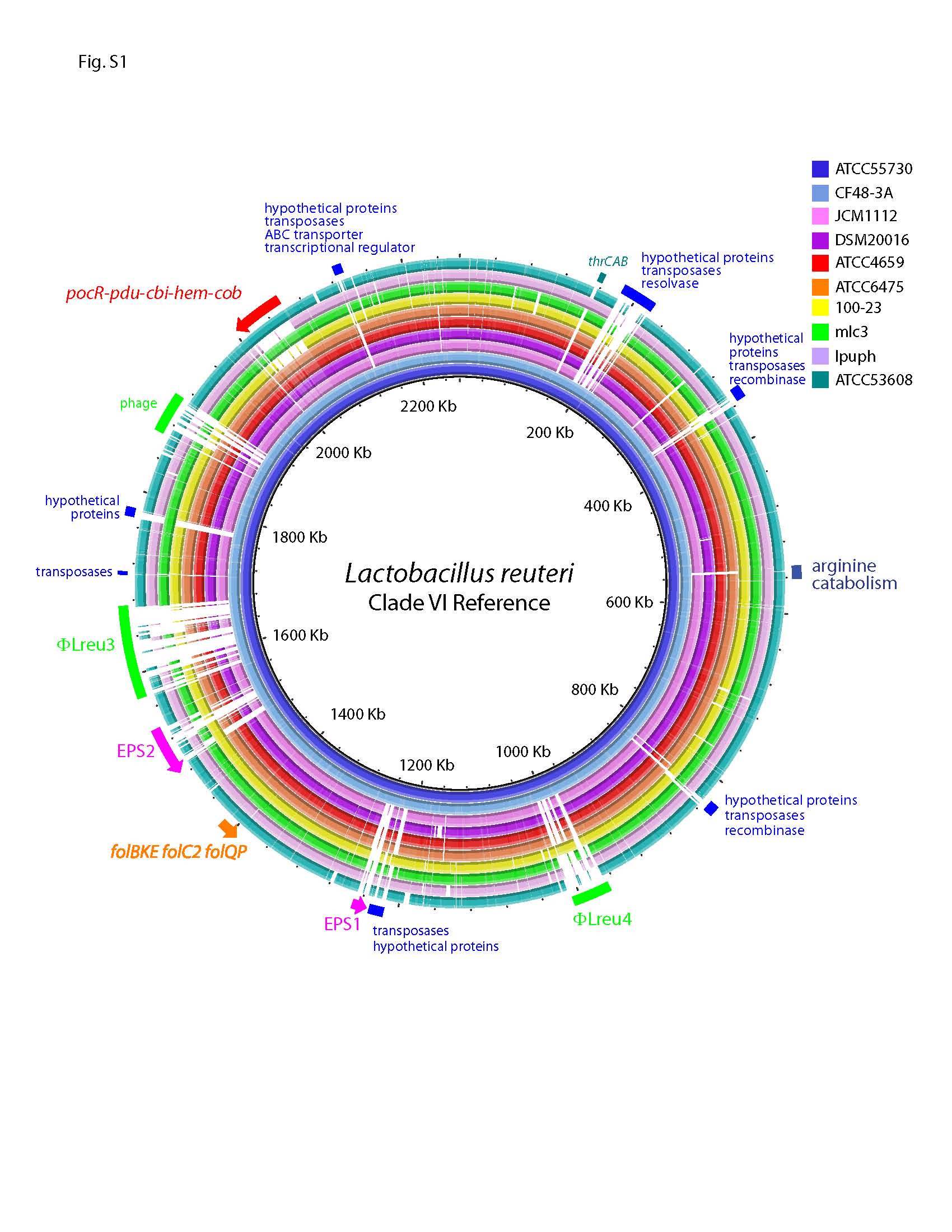

Supplement: Supplementary Data [file supp_evu137_Fig_S1_GBEr.jpg]

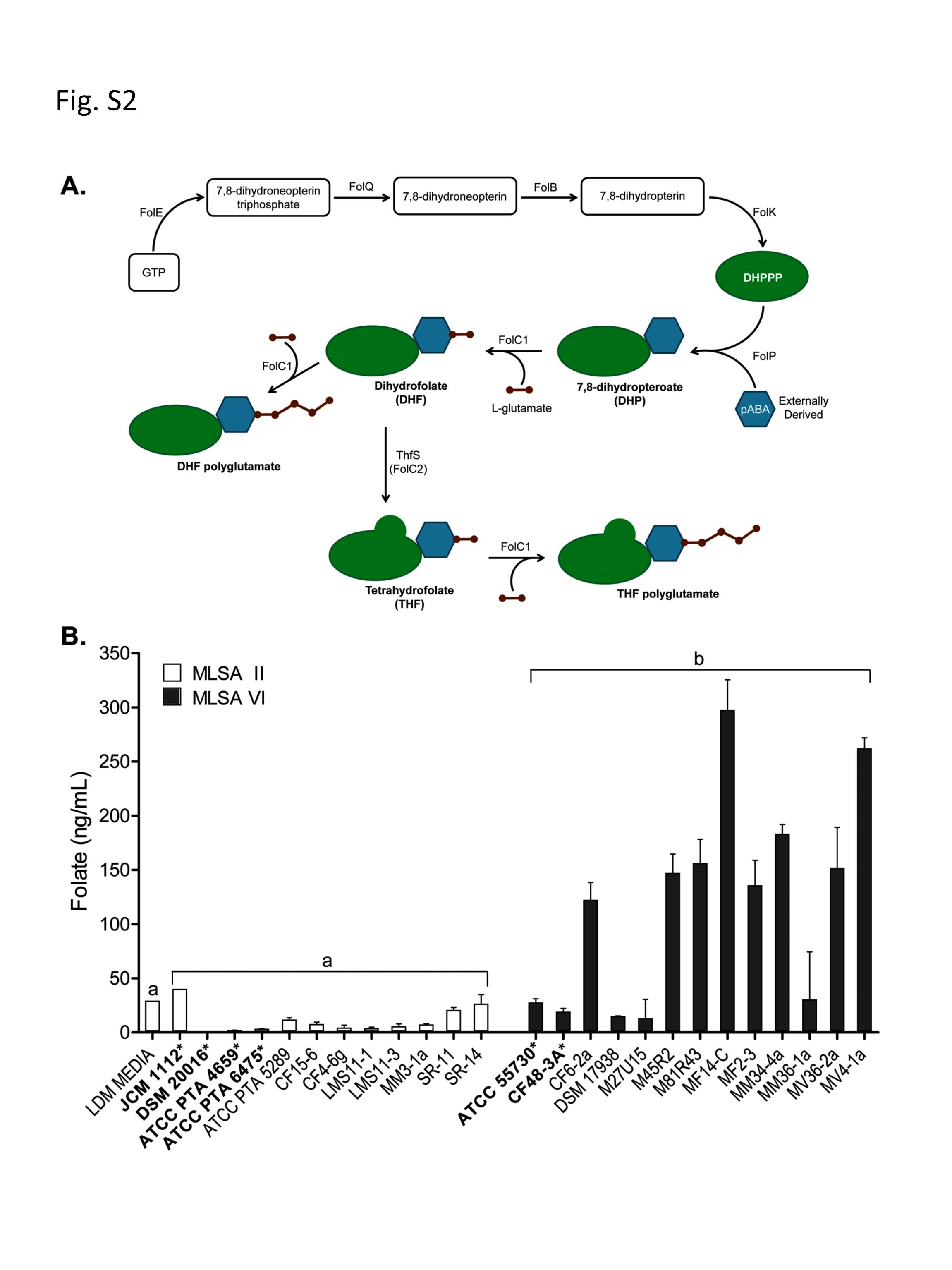

Supplement: Supplementary Data [file supp_evu137_Fig_S2_GBEr.jpg]

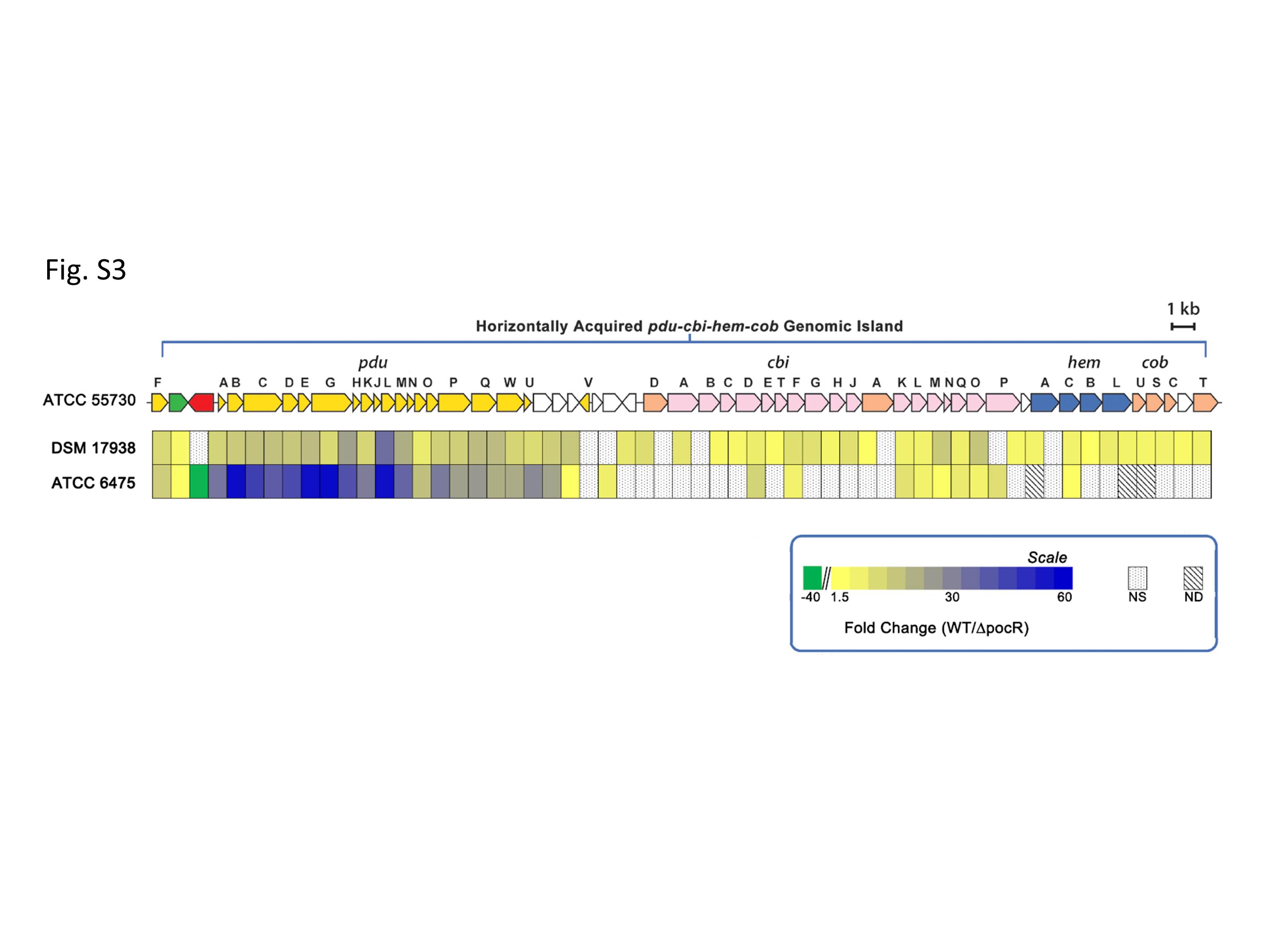

Supplement: Supplementary Data [file supp_evu137_Fig_S3_GBEr.jpg]
