# Supplementary material for: From Prediction to Function Using Evolutionary Genomics: Human-Specific Ecotypes of Lactobacillus reuteri Have Diverse Probiotic Functions
Source: Genome Biol Evol. 2014 Jun 19;6(7):1772–89. doi: 10.1093/gbe/evu137 (PMC4122935; doi:10.1093/gbe/evu137)
Supplement: Supplementary Data [file supp_evu137_Table_S1_GBEr.docx]

**Supplementary Table S1. Primers used in this study.**

| Primer Name | Sequence (5’-3’) | Target Gene | Reference |
| --- | --- | --- | --- |
| RB1883F2 5’BHI | TGACGGATCCTAAGTGTGACTGGCACTGTTGTAGATTGTAATACGC | *pocR* (DSM 17938) | This Study |
| RB1883R2 3’ERI | TGACGATTCGTCAACATCAATATGTGAGGCATCAAG | *pocR* (DSM 17938) | This Study |
| hdcP F3 | TGGACATTGTGCATATTCCTATTCC | *hdcP* | This Study |
| hdcP R | GCGCTTCCTATTCCTTACAATCC | *hdcP* | This Study |
| hdcA F | GGATTGTAAGGAATAGGAAGCGC | *hdcA* | This Study |
| hdcA R | GCTCCACATCCACCTACCTTA | *hdcA* | This Study |
| hdcB F | GCTGTTTGTGTACCACGATGTA | *hdcB* | This Study |
| hdcB R | CCTAAGGTAAAGTGTTCCACCG | *hdcB* | This Study |
| hisS F | ATTTCGTGATGGGGAGCGTT | *hisS2* | This Study |
| hisS R | TGCGGATAATTGAAGCCCATTA | *hisS2* | This Study |
