# Supplementary material for: From Prediction to Function Using Evolutionary Genomics: Human-Specific Ecotypes of Lactobacillus reuteri Have Diverse Probiotic Functions
Source: Genome Biol Evol. 2014 Jun 19;6(7):1772–89. doi: 10.1093/gbe/evu137 (PMC4122935; doi:10.1093/gbe/evu137)
Supplement: Supplementary Data [file supp_evu137_Table_S2_GBEr.docx]

**Supplementary Table S2a. Prophage** φ**Lreu1 Gene Annotations^a^**

| **New Annotation Coordinates** | | | | |
| --- | --- | --- | --- | --- |
| **Gene Start** | **Gene Stop** | **Strand** | **Protein  (aa)** | **Protein Function Prediction** |
| 878463 | 879728 | - | 421 | integrase/recombinase |
| 880043 | 880195 | + | 50 | transposase |
| 880196 | 881053 | - | 285 | hypothetical protein |
| 881074 | 881595 | - | 173 | hypothetical protein |
| 881609 | 882013 | - | 134 | conserved hypothetical *Lactobacillus* phage protein |
| 882067 | 882471 | - | 134 | conserved hypothetical protein |
| 882488 | 882823 | - | 111 | transcriptional regulator/phage repressor |
| 882984 | 883199 | + | 71 | transcriptional regulator/phage repressor |
| 883218 | 883991 | + | 257 | phage antirepressor |
| 884003 | 884131 | + | 42 | hypothetical protein |
| 884131 | 884253 | + | 40 | hypothetical protein |
| 884267 | 884470 | + | 67 | hypothetical protein |
| 884467 | 884715 | + | 82 | possible transcriptional regulator |
| 884693 | 884896 | + | 67 | hypothetical protein |
| 884911 | 885087 | + | 58 | hypothetical protein |
| 885087 | 885359 | + | 90 | transcriptional regulator |
| 885352 | 886281 | + | 309 | RecT family recombinase |
| 886265 | 887089 | + | 274 | conserved hypothetical protein |
| 887094 | 887951 | + | 285 | phage replication protein |
| 887944 | 888273 | + | 109 | hypothetical protein |
| 888270 | 888641 | + | 123 | hypothetical protein |
| 888638 | 888730 | + | 30 | hypothetical protein |
| 888717 | 888995 | + | 92 | hypothetical protein |
| 888992 | 889180 | + | 62 | hypothetical protein |
| 889180 | 889332 | + | 50 | hypothetical protein |
| 889376 | 889570 | + | 64 | hypothetical protein |
| 889570 | 889830 | + | 86 | hypothetical protein |
| 889830 | 890171 | + | 113 | hypothetical protein |
| 890171 | 890359 | + | 62 | hypothetical protein |
| 890367 | 890627 | + | 86 | hypothetical protein |
| 890627 | 890938 | + | 103 | hypothetical protein |
| 891001 | 891144 | + | 47 | hypothetical protein |
| 891226 | 891648 | + | 140 | possible phage recombinase/resolvase |
| 891661 | 891945 | + | 94 | conserved hypothetical protein |
| 892596 | 892802 | + | 68 | hypothetical protein |
| 892890 | 893558 | + | 222 | conserved hypothetical protein |
| 893558 | 893983 | + | 141 | conserved hypothetical protein |
| 893997 | 894776 | + | 259 | possible ParB family nuclease |
| 894793 | 895296 | + | 167 | hypothetical protein |
| 895271 | 896566 | + | 431 | phage terminase, large subunit |
| 896566 | 898227 | + | 553 | phage portal protein |
| 898227 | 899177 | + | 316 | phage head morphogenesis protein |
| 899188 | 899433 | + | 81 | hypothetical protein |
| 899563 | 900216 | + | 217 | phage scaffold protein |
| 900231 | 901307 | + | 358 | phage capsid protein |
| 901320 | 901685 | + | 121 | conserved hypothetical *Lactobacillus* phage protein |
| 901685 | 901999 | + | 104 | conserved hypothetical *Lactobacillus* phage protein |
| 901989 | 902549 | + | 186 | phage head-tail joining protein |
| 902558 | 902968 | + | 136 | conserved hypothetical *Lactobacillus* phage protein |
| 902971 | 903618 | + | 215 | phage tail protein |
| 903638 | 904183 | + | 181 | conserved hypothetical *Lactobacillus* phage protein |
| 904276 | 904455 | + | 59 | hypothetical protein |
| 904459 | 908109 | + | 1216 | phage tail tape measure protein |
| 908106 | 908993 | + | 295 | conserved hypothetical phage protein |
| 908990 | 911221 | + | 743 | M23 family peptidase/possible phage lysin |
| 911205 | 914786 | + | 1193 | conserved hypothetical protein |
| 914779 | 915051 | + | 90 | hypothetical protein |
| 915097 | 915366 | + | 89 | conserved hypothetical *Lactobacillus* phage protein |
| 915379 | 916524 | + | 381 | probable lipase |
| 916527 | 917636 | + | 369 | conserved hypothetical *Lactobacillus* phage protein |
| 917648 | 918052 | + | 134 | hypothetical protein |
| 918045 | 918164 | + | 39 | hypothetical protein |
| 918203 | 918754 | + | 183 | hypothetical protein |
| 918793 | 919962 | + | 389 | hypothetical protein |
| 919977 | 920426 | + | 149 | phage holin protein |
| 920416 | 921615 | + | 399 | N-acetylmuramoyl-L-alanine amidase |
| 921687 | 921854 | - | 55 | conserved hypothetical protein |
| 921867 | 923396 | - | 509 | glucokinase |
| 923624 | 924157 | + | 177 | GNAT family acetyltransferase |
| 924421 | 924525 | - | 34 | hypothetical protein |
| 924665 | 924874 | - | 69 | *Lactobacillus* conserved hypothetical protein |
| 925668 | 927581 | + | 637 | amidase |
| 927663 | 927938 | + | 91 | *Lactobacillus* conserved hypothetical protein |
| 927999 | 928490 | - | 163 | conserved hypothetical *Lactobacillus* phage protein |
| 928800 | 930332 | + | 510 | type I restriction modification system DNA methyltransferase subunit HsdM |
| 930322 | 930909 | + | 195 | type I restriction modification system DNA specificity subunit HsdS |
| 930830 | 931465 | - | 211 | type I restriction modification system DNA specificity subunit HsdS |
| 931508 | 932482 | + | 324 | integrase |

^a^Coordinates are based on the nucleotide sequence of JCM 1112 (GenBank NC_01609.1).

**Supplementary Table S2b. Prophage φLreu2 Gene Annotations^a^**

| **New Annotation Coordinates** | | | | |
| --- | --- | --- | --- | --- |
| **Gene Start** | **Gene Stop** | **Strand** | **Protein (aa)** | **Protein Function Prediction** |
| 1239954 | 1241084 | + | 376 | phage integrase |
| 1239634 | 1239951 | + | 105 | *Lactobacillus* conserved hypothetical protein |
| 1238962 | 1239471 | + | 169 | conserved hypothetical protein |
| 1238393 | 1238833 | + | 146 | conserved hypothetical protein |
| 1237943 | 1238380 | + | 145 | phage repressor |
| 1237561 | 1237764 | - | 67 | phage repressor |
| 1236739 | 1237512 | - | 257 | phage antirepressor |
| 1236449 | 1236727 | - | 92 | *Lactobacillus* conserved hypothetical protein |
| 1236326 | 1236448 | - | 40 | hypothetical protein |
| 1236183 | 1236314 | - | 43 | hypothetical protein |
| 1235663 | 1236169 | + | 168 | hypothetical protein |
| 1235425 | 1235610 | - | 61 | hypothetical protein |
| 1235159 | 1235389 | - | 76 | hypothetical protein |
| 1234177 | 1235166 | - | 329 | phage RecT family recombinase |
| 1233369 | 1234193 | - | 274 | conserved hypothetical protein |
| 1232507 | 1233364 | - | 285 | phage replication protein |
| 1232185 | 1232514 | - | 109 | hypothetical protein |
| 1231817 | 1232188 | - | 123 | hypothetical protein |
| 1231728 | 1231820 | - | 30 | hypothetical protein |
| 1231463 | 1231741 | - | 92 | hypothetical protein |
| 1231278 | 1231466 | - | 62 | hypothetical protein |
| 1231126 | 1231278 | - | 50 | hypothetical protein |
| 1230888 | 1231082 | - | 64 | hypothetical protein |
| 1230628 | 1230888 | - | 86 | hypothetical protein |
| 1230287 | 1230628 | - | 113 | hypothetical protein |
| 1230099 | 1230287 | - | 62 | hypothetical protein |
| 1229831 | 1230091 | - | 86 | hypothetical protein |
| 1229520 | 1229831 | - | 103 | hypothetical protein |
| 1229314 | 1229457 | - | 47 | hypothetical protein |
| 1228810 | 1229232 | - | 140 | *Lactobacillus* conserved hypothetical protein |
| 1228513 | 1228797 | - | 94 | hypothetical protein |
| 1227062 | 1227904 | - | 280 | hypothetical protein |
| 1226331 | 1226789 | - | 152 | IS*200* family transposase |
| 1226026 | 1226172 | - | 48 | Lactobacillus conserved hypothetical protein |
| 1225492 | 1225671 | - | 59 | Lactobacillus conserved hypothetical protein |
| 1224888 | 1225424 | - | 178 | phage endonuclease |
| 1224270 | 1224746 | - | 158 | phage terminase small subunit protein |
| 1223810 | 1224247 | + | 145 | hypothetical protein |
| 1223640 | 1223813 | + | 57 | hypothetical protein |
| 1221685 | 1223574 | - | 629 | phage terminase large subunit protein |
| 1220308 | 1221498 | - | 396 | phage portal protein |
| 1219590 | 1220321 | - | 243 | S14 family ClpP protease |
| 1218425 | 1219600 | - | 391 | phage major capsid protein |
| 1218037 | 1218405 | - | 122 | phage DNA packaging protein |
| 1217718 | 1218068 | - | 116 | phage head-tail joining/adaptor protein |
| 1217300 | 1217716 | - | 138 | phage head-tail joining protein |
| 1216920 | 1217303 | - | 127 | phage tail protein |
| 1216205 | 1216915 | - | 236 | phage tail protein |
| 1215757 | 1216143 | - | 128 | *Lactobacillus* conserved hypothetical protein |
| 1215533 | 1215682 | - | 49 | hypothetical protein |
| 1211701 | 1215531 | - | 1276 | phage tail tape measure protein |
| 1210841 | 1211686 | - | 281 | phage tail protein |
| 1209082 | 1210827 | - | 581 | glycoside hydrolase/peptidase/lysin |
| 1205055 | 1209134 | - | 1359 | hypothetical protein |
| 1204820 | 1205062 | - | 80 | hypothetical protein |
| 1204508 | 1204777 | - | 89 | conserved hypothetical *Lactobacillus* phage protein |
| 1203350 | 1204495 | - | 381 | conserved hypothetical *Lactobacillus* phage protein |
| 1202238 | 1203347 | - | 369 | conserved hypothetical *Lactobacillus* phage protein |
| 1201822 | 1202226 | - | 134 | hypothetical protein |
| 1201710 | 1201829 | - | 39 | hypothetical protein |
| 1201120 | 1201671 | - | 183 | hypothetical protein |
| 1199993 | 1201081 | - | 362 | hypothetical protein |
| 1199652 | 1199978 | - | 108 | hypothetical protein |
| 1199284 | 1199655 | - | 123 | hypothetical protein |
| 1198095 | 1199294 | - | 399 | N-acetylmuramoyl-L-alanine amidase |
| 1197209 | 1197328 | + | 39 | hypothetical protein |
| 1196399 | 1196857 | - | 152 | IS*200* family transposase |

^a^Coordinates are based on the nucleotide sequence of JCM 1112 (GenBank NC_01609.1).

**Supplementary Table S2c. Prophage φLreu3 Gene Annotations^a^**

| **New Annotation Coordinates** | | | | |
| --- | --- | --- | --- | --- |
| **Gene Start** | **Gene Stop** | **Strand** | **Protein (aa)** | **Protein Function Prediction** |
| 462928 | 463611 | + | 227 | integrase |
| 463616 | 464374 | + | 252 | ISChy4 transposase |
| 464301 | 464597 | - | 98 | hypothetical protein HMPREF0538_20464 |
| 464600 | 465334 | - | 244 | type II restriction-modification system modification subunit |
| 465378 | 465590 | - | 70 | hypothetical protein HMPREF0538_20466 |
| 465619 | 468228 | - | 869 | hypothetical protein HMPREF0538_20467 |
| 468234 | 469532 | - | 432 | hypothetical protein HMPREF0538_20468 |
| 469910 | 470368 | + | 152 | hypothetical protein HMPREF0538_20469 |
| 470451 | 470654 | + | 67 | hypothetical protein HMPREF0538_20470 |
| 470638 | 470955 | + | 105 | hypothetical protein HMPREF0538_20471 |
| 470952 | 472091 | + | 379 | *Lactobacillus* conserved phage protein |
| 472069 | 472644 | + | 191 | phage-associated protein |
| 472700 | 474634 | + | 644 | DNA polymerase |
| 474701 | 475105 | + | 134 | hypothetical protein HMPREF0538_20475 |
| 475108 | 477363 | + | 751 | P4 family phage protein |
| 477411 | 477857 | + | 148 | VRR-NUC domain protein |
| 477838 | 479193 | + | 451 | SNF2 domain protein |
| 479190 | 479657 | + | 155 | probable restriction endonuclease |
| 479806 | 480183 | + | 125 | HNH endonuclease domain protein |
| 480303 | 480845 | + | 180 | hypothetical protein HMPREF0538_20481 |
| 480845 | 482071 | + | 408 | DNA (cytosine-5-)-methyltransferase |
| 482144 | 482764 | + | 206 | hypothetical protein HMPREF0538_20483 |
| 482767 | 482973 | + | 68 | hypothetical protein HMPREF0538_20484 |
| 483104 | 484072 | + | 322 | transposase |
| 484096 | 485715 | + | 539 | prophage protein |
| 485744 | 487009 | + | 421 | HK97 family portal protein |
| 487006 | 487677 | + | 223 | S14 family peptidase ClpP |
| 487696 | 488874 | + | 392 | HK97 family phage protein |
| 488891 | 489169 | + | 92 | hypothetical protein HMPREF0538_20490 |
| 489169 | 489552 | + | 127 | bacteriophage head-tail adaptor |
| 489539 | 489952 | + | 137 | holin |
| 489949 | 490098 | + | 49 | hypothetical protein HMPREF0538_20493 |
| 490120 | 490239 | + | 39 | hypothetical protein HMPREF0538_20494 |
| 490306 | 490827 | + | 173 | lysozyme |
| 491094 | 491327 | + | 77 | hypothetical protein HMPREF0538_20496 |
| 491363 | 493024 | + | 553 | integrase/recombinase |
| 493017 | 494594 | + | 525 | Integrase/recombinase |
| 494764 | 495615 | + | 283 | ABC transporter ATP-binding protein |
| 495578 | 496468 | + | 296 | ABC transporter |
| 496471 | 496923 | + | 150 | LytR/AlgR family transcriptional regulator |
| 496925 | 497311 | + | 128 | hypothetical protein HMPREF0538_20502 |
| 497439 | 497561 | - | 40 | hypothetical protein HMPREF0538_20503 |
| 497928 | 498164 | - | 78 | hypothetical protein HMPREF0538_20504 |
| 498189 | 501305 | - | 1038 | type I site-specific deoxyribonuclease |
| 501360 | 502517 | - | 385 | type I restriction/modification specificity protein |
| 502514 | 503482 | - | 322 | integrase/recombinase |
| 503550 | 504209 | + | 219 | type I restriction-modification system specificity subunit |
| 504206 | 504736 | - | 176 | hypothetical protein HMPREF0538_20509 |
| 504726 | 506264 | - | 512 | type I restriction-modification system DNA-methyltransferase |
| 506261 | 506482 | - | 73 | hypothetical protein HMPREF0538_20511 |
| 506494 | 509106 | - | 870 | hypothetical protein HMPREF0538_20512 |
| 509111 | 510496 | - | 461 | hypothetical protein HMPREF0538_20513 |
| 510807 | 511325 | + | 172 | sigma-70 family protein |
| 511407 | 511628 | + | 73 | hypothetical protein HMPREF0538_20515 |
| 511612 | 511947 | + | 111 | hypothetical protein HMPREF0538_20516 |
| 511944 | 513080 | + | 378 | phage protein |
| 513061 | 513636 | + | 191 | hypothetical protein HMPREF0538_20518 |
| 513692 | 515626 | + | 644 | DNA-directed DNA polymerase |
| 515713 | 516099 | + | 128 | hypothetical protein HMPREF0538_20520 |
| 516102 | 518357 | + | 751 | P4 family prophage protein |
| 518405 | 518851 | + | 148 | VRR-NUC domain protein |
| 518832 | 520187 | + | 451 | SNF2 domain protein |
| 520184 | 520651 | + | 155 | probable restriction endonuclease |
| 520799 | 521176 | + | 125 | HNH endonuclease domain protein |
| 521299 | 521841 | + | 180 | hypothetical protein HMPREF0538_20526 |
| 521841 | 523070 | + | 409 | DNA (cytosine-5-)-methyltransferase |
| 523144 | 523776 | + | 210 | hypothetical protein HMPREF0538_20528 |
| 523769 | 523975 | + | 68 | hypothetical protein HMPREF0538_20529 |
| 524041 | 525642 | + | 533 | prophage protein |
| 525670 | 525831 | + | 53 | hypothetical protein HMPREF0538_20531 |
| 525972 | 526133 | - | 53 | lipoprotein |
| 526190 | 527446 | + | 418 | HK97 family portal protein |
| 527443 | 528105 | + | 220 | S14 family peptidase ClpP |
| 528126 | 529304 | + | 392 | HK97 family major capsid protein |
| 529317 | 529595 | + | 92 | hypothetical protein HMPREF0538_20536 |
| 529596 | 529976 | + | 126 | bacteriophage head-tail adaptor |
| 529966 | 530388 | + | 140 | holin |
| 530486 | 530674 | + | 62 | hypothetical protein HMPREF0538_20539 |
| 530736 | 532289 | + | 517 | phage integrase/recombinase |
| 532276 | 532680 | + | 134 | phage integrase/recombinase |
| 532667 | 534253 | + | 528 | phage integrase/recombinase |

^a^Coordinates are based on the nucleotide sequence of ATCC 55730 (GenBank NC_015697).

**Supplementary Table S2d. Prophage φLreu4 Gene Annotations^a^**

| **New Annotation Coordinates** | | | | |
| --- | --- | --- | --- | --- |
| **Gene Start** | **Gene Stop** | **Strand** | **Protein (aa)** | **Protein Function Prediction** |
| 2099733 | 2099939 | + | 68 | hypothetical protein HMPREF0538_22063 |
| 2099951 | 2100718 | + | 255 | phage antirepressor |
| 2100730 | 2100858 | + | 42 | hypothetical protein HMPREF0538_22065 |
| 2100858 | 2100980 | + | 40 | hypothetical protein HMPREF0538_22066 |
| 2100995 | 2101201 | + | 68 | DNA-binding protein |
| 2101198 | 2101377 | + | 59 | hypothetical protein HMPREF0538_22068 |
| 2101413 | 2101643 | + | 76 | hypothetical protein HMPREF0538_22069 |
| 2101636 | 2101908 | + | 90 | 6-phospho-beta-glucosidase |
| 2101912 | 2102664 | + | 250 | Erf family protein |
| 2102667 | 2103347 | + | 226 | hypothetical protein HMPREF0538_22072 |
| 2103337 | 2103765 | + | 142 | single-strand binding protein |
| 2103778 | 2104608 | + | 276 | phage protein |
| 2104624 | 2105421 | + | 265 | DNA replication protein |
| 2105424 | 2105576 | + | 50 | chaperone DnaJ |
| 2105580 | 2105840 | + | 86 | hypothetical protein HMPREF0538_22077 |
| 2105830 | 2106351 | + | 173 | hypothetical protein HMPREF0538_22078 |
| 2106364 | 2106921 | + | 185 | hypothetical protein HMPREF0538_22079 |
| 2106998 | 2107447 | + | 149 | hypothetical protein HMPREF0538_22080 |
| 2107440 | 2107718 | + | 92 | hypothetical protein HMPREF0538_22081 |
| 2107715 | 2108086 | + | 123 | oxygen-independent coproporphyrinogen III oxidase |
| 2108101 | 2109231 | - | 376 | IS*30* family transposase |
| 2109293 | 2109490 | + | 65 | XRE family transcriptional regulator |
| 2109480 | 2109722 | + | 80 | hypothetical protein HMPREF0538_22085 |
| 2109691 | 2110077 | + | 128 | hypothetical protein HMPREF0538_22086 |
| 2110190 | 2110384 | + | 64 | hypothetical protein HMPREF0538_22087 |
| 2110488 | 2110631 | + | 47 | hypothetical protein HMPREF0538_22088 |
| 2110663 | 2110854 | + | 63 | hypothetical protein HMPREF0538_22089 |
| 2110964 | 2111479 | + | 171 | hypothetical protein HMPREF0538_22090 |
| 2112304 | 2112783 | + | 159 | XRE family transcriptional regulator |
| 2112773 | 2113207 | + | 144 | hypothetical protein HMPREF0538_22092 |
| 2113753 | 2113932 | + | 59 | probable phage-associated protein |
| 2113925 | 2114203 | + | 92 | hypothetical protein HMPREF0538_22094 |
| 2114447 | 2114617 | + | 56 | hypothetical protein HMPREF0538_22095 |
| 2114643 | 2114882 | - | 79 | hypothetical protein HMPREF0538_22096 |
| 2114838 | 2115695 | + | 285 | HNH endonuclease domain protein |
| 2115879 | 2116346 | + | 155 | phage terminase small subunit |
| 2116343 | 2118052 | + | 569 | phage terminase large subunit |
| 2118033 | 2118395 | + | 120 | hypothetical protein HMPREF0538_22100 |
| 2118558 | 2119817 | + | 419 | phage portal protein |
| 2119756 | 2120409 | + | 217 | phage head maturation protease |
| 2120410 | 2121546 | + | 378 | phage protein |
| 2121688 | 2122101 | + | 137 | phage protein |
| 2122004 | 2122357 | + | 117 | hypothetical protein HMPREF0538_22105 |
| 2122341 | 2122715 | + | 124 | phage protein |
| 2122712 | 2123131 | + | 139 | phage protein |
| 2123131 | 2123760 | + | 209 | phage major tail protein |
| 2123813 | 2124154 | + | 113 | glutamyl-tRNA synthetase |
| 2124163 | 2124333 | + | 56 | hypothetical protein HMPREF0538_22110 |
| 2124350 | 2128852 | + | 1500 | TP901 family phage tail tape measure protein |
| 2128856 | 2129698 | + | 280 | hypothetical protein HMPREF0538_22112 |
| 2129773 | 2132241 | + | 822 | phage lysin |
| 2132254 | 2132616 | + | 120 | hypothetical protein HMPREF0538_22114 |
| 2132616 | 2134490 | + | 624 | phage minor head protein |
| 2134502 | 2135287 | + | 261 | hypothetical protein HMPREF0538_22116 |
| 2135302 | 2135700 | + | 132 | hypothetical protein HMPREF0538_22117 |
| 2135944 | 2136324 | + | 126 | hypothetical protein HMPREF0538_22118 |
| 2136485 | 2136961 | + | 158 | hypothetical protein HMPREF0538_22119 |
| 2136974 | 2137900 | + | 308 | endolysin |
| 2138047 | 2138205 | + | 52 | hypothetical protein HMPREF0538_22121 |

^a^Coordinates are based on the nucleotide sequence of ATCC 55730 (GenBank NC_015697).
