# Supplementary material for: From Prediction to Function Using Evolutionary Genomics: Human-Specific Ecotypes of Lactobacillus reuteri Have Diverse Probiotic Functions
Source: Genome Biol Evol. 2014 Jun 19;6(7):1772–89. doi: 10.1093/gbe/evu137 (PMC4122935; doi:10.1093/gbe/evu137)
Supplement: Supplementary Data [file supp_evu137_Table_S3_GBEr.docx]

**Supplementary Table S3. Arginine Catabolism Gene Cluster Annotations^a^**

| **New Annotation Coordinates** | | | | |
| --- | --- | --- | --- | --- |
| **Gene Start** | **Gene Stop** | **Strand** | **Protein (aa)** | **Protein Function Prediction** |
| 484793 | 485251 | - | 152 | IS*200* family transposase |
| 485472 | 486311 | - | 279 | transcriptional regulator |
| 486564 | 487571 | + | 335 | ornithine carbamoyltransferase ArcB |
| 487588 | 488520 | + | 310 | carbamate kinase ArcC |
| 488564 | 489478 | - | 304 | DMT drug/metabolite transporter |
| 489562 | 490143 | - | 193 | M10 family zinc metallopeptidase |
| 490419 | 490787 | + | 122 | probable secreted cupredoxin domain protein |
| 490787 | 491074 | + | 95 | probable cupredoxin domain protein |
| 491074 | 493011 | + | 645 | P-type ATPase superfamily copper transporter |
| 493130 | 493606 | + | 158 | *Lactobacillus* conserved hypothetical protein |
| 493835 | 494164 | - | 109 | *Lactobacillus* conserved hypothetical protein |
| 494395 | 495783 | + | 462 | CPA1 family monovalent Na+/H+ antiporter |
| 496429 | 496611 | - | 60 | conserved hypothetical protein |
| 496613 | 496789 | - | 58 | hypothetical protein |
| 497061 | 497399 | - | 112 | transposase |
| 497426 | 498307 | - | 293 | transposase |
| 499483 | 499848 | - | 121 | hypothetical protein |
| 499845 | 499976 | - | 43 | hypothetical protein |
| 500400 | 500801 | - | 133 | *Lactobacillus* conserved hypothetical protein |
| 500818 | 501219 | - | 133 | hypothetical protein |
| 501268 | 502227 | - | 319 | hypothetical protein |
| 502244 | 502468 | - | 74 | hypothetical protein |
| 502468 | 502938 | - | 156 | hypothetical protein |
| 503095 | 503859 | - | 254 | possible ribonuclease H |
| 503856 | 504455 | - | 199 | conserved hypothetical protein |
| 504681 | 505274 | - | 197 | hypothetical protein |
| 505465 | 506484 | + | 339 | beta-lactamase/penicillin binding protein |
| 506692 | 507924 | + | 410 | arginine deiminase ArcA |
| 508035 | 508496 | + | 153 | ArgR family transcriptional regulator |
| 508517 | 509938 | + | 473 | APC family amino acid polyamine organocation transporter |
| 509996 | 511393 | + | 466 | APC family amino acid polyamine organocation transporter |

^a^Coordinates are based on the nucleotide sequence of JCM 1112 (GenBank NC_01609.1).
