# Supplementary material for: From Prediction to Function Using Evolutionary Genomics: Human-Specific Ecotypes of Lactobacillus reuteri Have Diverse Probiotic Functions
Source: Genome Biol Evol. 2014 Jun 19;6(7):1772–89. doi: 10.1093/gbe/evu137 (PMC4122935; doi:10.1093/gbe/evu137)
Supplement: Supplementary Data [file supp_evu137_Table_S4_GBEr.docx]

**Supplementary Table S4. Folate biosynthesis gene annotations^*^**

| **Gene** | **Protein Function Prediction** | **Strand** | **Protein (aa)** | **ATCC 55730^a^** | | **JCM 1112^b^** | |
| --- | --- | --- | --- | --- | --- | --- | --- |
|  |  |  |  | **Start** | **Stop** | **Start** | **Stop** |
| *folC1* | folypolyglutamate synthase | + | 437 | 1764152 | 1765465 | 578388 | 579701 |
| *folA* | dihydrofolate reductase | + | 162 | 2068933 | 2069421 | 853517 | 854005 |
| *folP* | dihydropteroate synthase | - | 387 | 277203 | 278366 | 1364163 | 1364975 |
| *folQ* | dihydroneopterin triphosphate diphosphatase | - | 195 | 278368 | 278955 | 1365328 | 1365915 |
| *thfS* (*folC2*) | tetrahydrofolate synthase | - | 419 | 278945 | 280204 | 1365905 | 1367164 |
| *folE* | GTP cyclohydrolase I | - | 192 | 280191 | 280769 | 1367151 | 1367729 |
| *folK* | 2-amino-4-hydroxy-6-hydroxymethyldihydropteridine diphosphokinase | - | 170 | 280751 | 281263 | 1367711 | 1368223 |
| *folB* | dihydroneopterin aldolase | - | 111 | 281266 | 281601 | 1368226 | 1368561 |

^*^Coordinates are based on the nucleotide sequence of ^a^ATCC 55730 (GenBank NC_015697) and ^b^JCM 1112 (GenBank NC_01609.1).
