# Supplementary material for: From Prediction to Function Using Evolutionary Genomics: Human-Specific Ecotypes of Lactobacillus reuteri Have Diverse Probiotic Functions
Source: Genome Biol Evol. 2014 Jun 19;6(7):1772–89. doi: 10.1093/gbe/evu137 (PMC4122935; doi:10.1093/gbe/evu137)
Supplement: Supplementary Data [file supp_evu137_Table_S5_GBEr.docx]

**Supplementary Table S5a. Clade VI *pdu-cbi-hem-cob* gene cluster annotations^a^**

| **Start** | **Stop** | **Strand** | **Protein (aa)** | **Gene** | **Protein Function Prediction** |
| --- | --- | --- | --- | --- | --- |
| 919827 | 920534 | - | 235 | *pduF* | MIP family glycerol uptake facilitator protein |
| 918921 | 919754 | - | 277 | *eutJ* | ethanolamine utilization protein |
| 917746 | 918840 | + | 364 | *pocR* | AraC-family transcriptional regulator |
| 917212 | 917490 | - | 92 | *pduA* | carbon dioxide concentrating mechanisms, carboxysome shell protein |
| 916398 | 917114 | - | 238 | *pduB* | propanediol utilization protein |
| 914697 | 916373 | - | 558 | *pduC* | glycerol dehydratase, large subunit |
| 913969 | 914679 | - | 236 | *pduD* | glycerol dehydratase, medium subunit |
| 913440 | 913955 | - | 171 | *pduE* | glycerol dehydratase, small subunit |
| 911565 | 913412 | - | 615 | *pduG* | propanediol dehydratase reactivation protein |
| 911219 | 911599 | - | 126 | *pduH* | propanediol dehydratase reactivation protein |
| 910642 | 911211 | - | 189 | *pduK* | propanediol utilization protein |
| 910342 | 910629 | - | 95 | *pduJ* | carbon dioxide concentrating mechanisms, carboxysome shell protein |
| 909675 | 910319 | - | 214 | *pduL* | propanediol utilization protein |
| 909141 | 909644 | - | 167 | *pduM* | propanediol utilization protein |
| 908881 | 909153 | - | 90 | *pduN* | carbon dioxide concentrating mechanisms, carboxysome shell protein |
| 908273 | 908860 | - | 195 | *pduO* | propanediol utilization protein |
| 907797 | 908270 | - | 157 | *pduO_bis_* | propanediol utilization protein |
| 906367 | 907794 | - | 475 | *pduP* | CoA-dependent propionaldehyde dehydrogenase |
| 905228 | 906367 | - | 379 | *pduQ* | propanediol dehydrogenase |
| 904017 | 905204 | - | 395 | *pduW* | acetate kinase |
| 903654 | 904001 | - | 115 | *pduU* | propanediol utilization protein |
| 902794 | 903588 | - | 264 |  | conserved hypothetical protein |
| 902014 | 902646 | - | 210 |  | phosphoglycerate mutase |
| 901457 | 902017 | - | 186 |  | hypothetical protein |
| 901036 | 901464 | + | 142 | *pduV* | propanediol utilization protein |
| 900479 | 900928 | - | 149 |  | flavodoxin |
| 899616 | 900464 | - | 282 |  | flavoprotein |
| 898973 | 899557 | + | 194 |  | putative ATP:cob(I)alamin adenosyltransferase |
| 897545 | 898633 | - | 362 | *cobD* | L-threonine-O-3-phosphate decarboxylase |
| 896184 | 897548 | - | 454 | *cbiA* | cobyrinic acid A, C-diamide synthase |
| 895228 | 896187 | - | 319 | *cbiB* | cobalamin biosynthesis protein |
| 894536 | 895222 | - | 228 | *cbiC* | precorrin-8X methylmutase |
| 893407 | 894564 | - | 385 | *cbiD* | cobalamin biosynthesis protein |
| 892811 | 893416 | - | 201 | *cbiE* | precorrin-6Y C5,15-methyltransferase |
| 892264 | 892818 | - | 184 | *cbiT* | precorrin-8W decarboxylase |
| 891488 | 892258 | - | 256 | *cbiF* | precorrin-4 C11-methyltransferase |
| 890430 | 891485 | - | 351 | *cbiG* | cobalamin biosynthesis protein |
| 889693 | 890418 | - | 241 | *cbiH* | precorrin-3B C17-methyltransferase |
| 888938 | 889696 | - | 252 | *cbiJ* | precorrin-6X reductase |
| 887554 | 888948 | - | 464 | *cobA* | uroporphyrinogen-III C-methyltransferase |
| 886785 | 887561 | - | 258 | *cbiK* | cobalt chelatase |
| 886081 | 886779 | - | 232 | *cbiL* | precorrin-2 C20-methyltransferase |
| 885335 | 886078 | - | 247 | *cbiM* | cobalamin biosynthesis protein |
| 885015 | 885338 | - | 107 | *cbiN* | cobalt ABC transporter permease component |
| 884312 | 884989 | - | 225 | *cbiQ* | cobalt ABC transporter permease component |
| 883493 | 884302 | - | 269 | *cbiO* | cobalt ABC transporter ATP-binding protein |
| 881886 | 883403 | - | 505 | *cbiP* | adenosylcobyric acid synthase |
| 881419 | 881889 | - | 156 | *cysG* | putative siroheme synthase |
| 880151 | 881416 | - | 421 | *hemA* | glutamyl-tRNA reductase |
| 879244 | 880161 | - | 305 | *hemC* | porphobilinogen deaminase |
| 878267 | 879238 | - | 323 | *hemB* | delta-aminolevulinic acid dehydratase |
| 876952 | 878283 | - | 443 | *hemL* | glutamate-1-semialdehyde 2,1-aminotransferase |
| 876300 | 876890 | - | 196 | *cobU* | adenosylocobinamide-phosphate guanylyltransferase |
| 875530 | 876291 | - | 253 | *cobS* | cobalamin-5’-phosphate synthase |
| 874943 | 875533 | - | 196 | *cobC* | alpha-ribazole-5’-phosphate phosphatase |
| 874219 | 874929 | - | 236 |  | type I site-specific deoxyribonuclease |
| 873165 | 874217 | - | 350 | *cobT* | nicotinate-nucleotide-dimethylbenzimidazole phosphoribosyltransferase |

^a^Coordinates are based on the nucleotide sequence of ATCC 55730 (GenBank NC_015697).

**Supplementary Table S5b. Clade II *pdu-cbi-hem-cob* gene cluster annotations^a^**

| **Start** | **Stop** | **Strand** | **Protein (aa)** | **Gene** | **Protein Function Prediction** |
| --- | --- | --- | --- | --- | --- |
| 1844734 | 1845441 | - | 235 | *pduF* | MIP family glycerol uptake facilitator protein |
| 1843816 | 1844649 | - | 277 | *eutJ* | ethanolamine utilization protein |
| 1842696 | 1843775 | + | 359 | *pocR* | AraC-family transcriptional regulator |
| 1842161 | 1842442 | - | 93 | *pduA* | carbon dioxide concentrating mechanisms, carboxysome shell protein |
| 1841347 | 1842063 | - | 238 | *pduB* | propanediol utilization protein |
| 1839646 | 1841322 | - | 558 | *pduC* | glycerol dehydratase, large subunit |
| 1838918 | 1839628 | - | 236 | *pduD* | glycerol dehydratase, medium subunit |
| 1838390 | 1838905 | - | 171 | *pduE* | glycerol dehydratase, small subunit |
| 1836509 | 1838359 | - | 616 | *pduG* | propanediol dehydratase reactivation protein |
| 1836163 | 1836546 | - | 127 | *pduH* | propanediol dehydratase reactivation protein |
| 1835586 | 1836155 | - | 189 | *pduK* | propanediol utilization protein |
| 1835283 | 1835573 | - | 96 | *pduJ* | carbon dioxide concentrating mechanisms, carboxysome shell protein |
| 1834610 | 1835254 | - | 214 | *pduL* | propanediol utilization protein |
| 1834075 | 1834578 | - | 167 | *pduM* | propanediol utilization protein |
| 1833815 | 1834093 | - | 92 | *pduN* | carbon dioxide concentrating mechanisms, carboxysome shell protein |
| 1833207 | 1833794 | - | 195 | *pduO* | propanediol utilization protein |
| 1832731 | 1833204 | - | 157 | *pduO_bis_* | propanediol utilization protein |
| 1831295 | 1832728 | - | 477 | *pduP* | CoA-dependent propionaldehyde dehydrogenase |
| 1830156 | 1831277 | - | 373 | *pduQ* | propanediol dehydrogenase |
| 1828948 | 1830132 | - | 394 | *pduW* | acetate kinase |
| 1828585 | 1828932 | - | 115 | *pduU* | propanediol utilization protein |
| 1827718 | 1828512 | - | 264 |  | conserved hypothetical protein |
| 1826977 | 1827621 | - | 214 |  | phosphoglycerate mutase |
| 1826423 | 1826980 | - | 185 |  | hypothetical protein |
| 1826002 | 1826430 | + | 142 | *pduV* | propanediol utilization protein |
| 1825446 | 1825895 | - | 149 |  | flavodoxin |
| 1824582 | 1825424 | - | 280 |  | flavoprotein |
| 1823968 | 1824534 | + | 188 |  | putative ATP:cob(I)alamin adenosyltransferase |
| 1822871 | 1823839 | - | 322 |  | transposase |
| 1821438 | 1822526 | - | 362 | *cobD* | L-threonine-O-3-phosphate decarboxylase |
| 1820074 | 1821438 | - | 454 | *cbiA* | cobyrinic acid A, C-diamide synthase |
| 1819118 | 1820077 | - | 319 | *cbiB* | cobalamin biosynthesis protein |
| 1818429 | 1819112 | - | 227 | *cbiC* | precorrin-8X methylmutase |
| 1817297 | 1818448 | - | 383 | *cbiD* | cobalamin biosynthesis protein |
| 1816698 | 1817300 | - | 200 | *cbiE* | precorrin-6Y C5,15-methyltransferase |
| 1816151 | 1816705 | - | 184 | *cbiT* | precorrin-8W decarboxylase |
| 1815373 | 1816134 | - | 253 | *cbiF* | precorrin-4 C11-methyltransferase |
| 1814315 | 1815370 | - | 351 | *cbiG* | cobalamin biosynthesis protein |
| 1813577 | 1814302 | - | 241 | *cbiH* | precorrin-3B C17-methyltransferase |
| 1812822 | 1813580 | - | 252 | *cbiJ* | precorrin-6X reductase |
| 1811438 | 1812832 | - | 464 | *cobA* | uroporphyrinogen-III C-methyltransferase |
| 1810666 | 1811445 | - | 259 | *cbiK* | cobalt chelatase |
| 1809951 | 1810664 | - | 237 | *cbiL* | precorrin-2 C20-methyltransferase |
| 1809221 | 1809967 | - | 248 | *cbiM* | cobalamin biosynthesis protein |
| 1808913 | 1809224 | - | 103 | *cbiN* | cobalt ABC transporter permease component |
| 1808215 | 1808892 | - | 225 | *cbiQ* | cobalt ABC transporter permease component |
| 1807400 | 1808203 | - | 267 | *cbiO* | cobalt ABC transporter ATP-binding protein |
| 1805820 | 1807325 | - | 501 | *cbiP* | adenosylcobyric acid synthase |
| 1805347 | 1805805 | - | 152 | *cysG* | putative siroheme synthase |
| 1804080 | 1805345 | - | 421 | *hemA* | glutamyl-tRNA reductase |
| 1803173 | 1804090 | - | 305 | *hemC* | porphobilinogen deaminase |
| 1802196 | 1803167 | - | 323 | *hemB* | delta-aminolevulinic acid dehydratase |
| 1800881 | 1802176 | - | 431 | *hemL* | glutamate-1-semialdehyde 2,1-aminotransferase |
| 1800228 | 1800818 | - | 196 | *cobU* | adenosylocobinamide-phosphate guanylyltransferase |
| 1799458 | 1800219 | - | 253 | *cobS* | cobalamin-5’-phosphate synthase |
| 1798871 | 1799461 | - | 196 | *cobC* | alpha-ribazole-5’-phosphate phosphatase |
| 1798124 | 1798834 | - | 236 |  | type I site-specific deoxyribonuclease |
| 1797070 | 1798140 | - | 356 | *cobT* | nicotinate-nucleotide-dimethylbenzimidazole phosphoribosyltransferase |

^a^Coordinates are based on the nucleotide sequence of JCM 1112 (GenBank NC_01609.1).
