# Supplementary material for: From Prediction to Function Using Evolutionary Genomics: Human-Specific Ecotypes of Lactobacillus reuteri Have Diverse Probiotic Functions
Source: Genome Biol Evol. 2014 Jun 19;6(7):1772–89. doi: 10.1093/gbe/evu137 (PMC4122935; doi:10.1093/gbe/evu137)
Supplement: Supplementary Data [file supp_evu137_Table_S8_GBEr.docx]

**Supplementary Table S8. *L. reuteri* Clade II Histidine Decarboxylase Gene Cluster^a^**

| **Start** | **Stop** | **Strand** | **Protein (aa)** | **Gene** | **Protein Function Prediction** |
| --- | --- | --- | --- | --- | --- |
| 1927408 | 1928862 | - | 484 | *hdcP* | APC family amino acid-polyamine-organocation transporter |
| 1926216 | 1927151 | - | 311 | *hdcA* | histidine decarboxylase |
| 1925451 | 1925975 | - | 174 | *hdcB* | conserved hypothetical protein HdcB |
| 1923821 | 1925122 | - | 433 | *hisRS2* | histidyl-tRNA synthase |

^a^Coordinates are based on the nucleotide sequence of JCM1112 (GenBank NC_010609).
